# An Autocrine Cytokine/JAK/STAT-Signaling Induces Kynurenine Synthesis in Multidrug Resistant Human Cancer Cells

**DOI:** 10.1371/journal.pone.0126159

**Published:** 2015-05-08

**Authors:** Ivana Campia, Ilaria Buondonno, Barbara Castella, Barbara Rolando, Joanna Kopecka, Elena Gazzano, Dario Ghigo, Chiara Riganti

**Affiliations:** 1 Department of Oncology, University of Torino, Torino, Italy; 2 Center for Experimental Research and Medical Studies (CeRMS), University of Torino, Torino, Italy; 3 Division of Hematology, University of Torino, Torino, Italy; 4 Department of Drug Science and Technology, University of Torino, Torino, Italy; University Hospital of Heidelberg, GERMANY

## Abstract

**Background:**

Multidrug resistant cancer cells are hard to eradicate for the inefficacy of conventional anticancer drugs. Besides escaping the cytotoxic effects of chemotherapy, they also bypass the pro-immunogenic effects induced by anticancer drugs: indeed they are not well recognized by host dendritic cells and do not elicit a durable anti-tumor immunity. It has not yet been investigated whether multidrug resistant cells have a different ability to induce immunosuppression than chemosensitive ones. We addressed this issue in human and murine chemosensitive and multidrug resistant cancer cells.

**Results:**

We found that the activity and expression of indoleamine 2,3-dioxygenase 1 (IDO1), which catalyzes the conversion of tryptophan into the immunosuppressive metabolite kynurenine, was higher in all the multidrug resistant cells analyzed and that IDO1 inhibition reduced the growth of drug-resistant tumors in immunocompetent animals. In chemoresistant cells the basal activity of JAK1/STAT1 and JAK1/STAT3 signaling was higher, the STAT3 inhibitor PIAS3 was down-regulated, and the autocrine production of STAT3-target and IDO1-inducers cytokines IL-6, IL-4, IL-1β, IL-13, TNF-α and CD40L, was increased. The disruption of the JAK/STAT signaling lowered the IDO1 activity and reversed the kynurenine-induced pro-immunosuppressive effects, as revealed by the restored proliferation of T-lymphocytes in STAT-silenced chemoresistant cells.

**Conclusions:**

Our work shows that multidrug resistant cells have a stronger immunosuppressive attitude than chemosensitive cells, due to the constitutive activation of the JAK/STAT/IDO1 axis, thus resulting chemo- and immune-evasive. Disrupting this axis may significantly improve the efficacy of chemo-immunotherapy protocols against resistant tumors.

## Introduction

Achieving a good chemotherapy efficacy and inducing a durable anti-tumor immune response are the main challenges of chemoimmunotherapy. Chemoresistance, in particular the simultaneous resistance towards different chemotherapeutic agents known as multidrug resistance (MDR), is one of the biggest problems encountered by chemotherapy [1]. MDR can be present at the diagnosis or induced by the selective pressure of chemotherapy; it often relies on the overexpression of ATP binding cassette (ABC) transporters responsible for the anticancer drug efflux, such as P-glycoprotein (Pgp), MDR related proteins (MRPs) and breast cancer resistance protein (BCRP). Together, they efflux both classical chemotherapeutic agents (e.g. anthracyclines, taxanes, Vinca alkaloids, epipodophyllotoxins, topotecan, methotrexate) and new targeted drugs (e.g. imatinib, dasatinib, lapatinib, gefitinib, sorafenib, erlotinib), limiting their cytotoxic effects [2].

Specific chemotherapeutic agents, such as anthracyclines and oxaliplatin, induce also pro-immunogenic effects, by inducing the translocation on the plasma membrane of specific “eat me” signals, like the chaperon calreticulin, which triggers the tumor cell phagocytosis and the subsequent activation of antitumor CD8^+^ T-lymphocytes [3]. This mechanism does not operate in cells overexpressing Pgp [4–6], which result at the same time chemo- and immune-resistant.

Moreover, tumor cells may evade the host immunosurveillance by suppressing the activity of the host immune system. A plethora of mechanisms mediate the tumor-induced immunosuppression, including: changes in tumor surface antigens; release of immunosuppressive cytokines in the tumor microenvironment; expansion of T-helper 2 lymphocytes, T-regulatory (Treg) cells, myeloid derived suppressor cells and type 2-tumor associated macrophages, which favor the tumor growth and impair the activity of anti-tumor populations, such as T-helper 1 lymphocytes, CD8^+^ T-lymphocytes, type 1-tumor associated macrophages, natural killer cells [7].

One of the strongest mediators of the tumor-induced immunosuppression is kynurenine, the product of tryptophan catabolism via tryptophan dioxygenase (TDO) [8] and indoleamine 2,3-dioxygenase enzymes (IDO1 and IDO2) [9], which are induced by interferon- γ (IFN-γ) [10, 11], nitric oxide (NO) [12] and iron [13]. Tryptophan is an essential amino acid for the proliferation and survival of CD8^+^ and CD4^+^ T-lymphocytes; moreover the increased kynurenine/tryptophan ratio severely compromises the efficiency of the host cellular immunity, because kynurenine inhibits the activation of T-lymphocytes [7, 14]. IDO1 is expressed in tumor-infiltrating dendritic cells [15] and in tumor stromal cells [16], and it has been found constitutively expressed or up-regulated in several tumor cells [14, 17]. An increased serum kynurenine/tryptophan ratio has been correlated to a faster progression of lung cancer [18] and the IDO positivity in tumor samples is usually associated with a poor clinical prognosis [19–21]. IDO1 over-expression supports tumor growth and progression of lung cancers [22], leading to hypothesize that kynurenine, besides its immunosuppressive effects, may directly enhance the tumor development.

We previously demonstrated that multidrug resistant cells are resistant to the immunogenic death operated by dendritic cells-mediated phagocytosis [4–6]. It has not been investigated whether multidrug resistant cells differ from chemosensitive ones also for the ability to induce immunosuppression: we found that multidrug resistant cells had a basally higher production of the immunosuppressive metabolite kynurenine than chemosensitive cells and we investigated the molecular basis of this phenotype.

## Materials and Methods

### Chemicals

The plasticware for cell cultures was from Falcon (Becton Dickinson, Franklin Lakes, NJ). The electrophoresis reagents were obtained from Bio-Rad Laboratories (Hercules, CA). The human recombinant IFN-γ was obtained from R&D Systems (Minneapolis, MN). 5-Br-brassinin was from Santa Cruz Biotechnology Inc. (Santa Cruz, CA). The protein content of cell lysates was assessed with the BCA kit from Sigma Chemicals Co (St. Louis, MO). When not otherwise specified, all the other reagents were purchased from Sigma Chemicals Co. Stock solutions of 3 mmol/L ferric nitrilotriacetate (FeNTA) were prepared by mixing 1 volume of 6 mmol/L nitrilotriacetic acid in 1 eq/L NaOH, and 1 volume of 6 mmol/L FeCl_3_ in 1 eq/L HCl; the pH was adjusted to neutrality with NaOH.

### Cells

The human chemosensitive non small cell lung cancer A549 cells were purchased from Istituto Zooprofilattico Sperimentale "Bruno Umbertini" (Brescia, Italy). The human chemosensitive colon cancer HT29 cells, the human chemosensitive chronic myelogenous leukemia K562 cells, the human chemosensitive mesothelial Met5A cells and the murine constitutively chemoresistant mammary JC cells were from ATCC (Manassas, VA) The resistant sublines A549/dx, HT29/dx, K562/dx were generated in our laboratory by culturing the above mentioned parental cells in a medium containing increasing concentrations of doxorubicin, used as a MDR inducer, as reported earlier [4]. The human constitutively chemoresistant malignant mesothelioma (HMM) cells were collected, after informed written consent from the patients, by the Biologic Bank of Malignant Mesothelioma (S.S. Antonio e Biagio Hospital, Alessandria, Italy), where the histological characterization was performed [23]. The experimental protocol (code: TASK3) was approved on 09/11/2011 by the Bioethics Committee (“Comitato Etico Interaziendale”) of the S.S. Antonio e Biagio Hospital, Alessandria, Italy. The cells were grown in the respective culture medium supplemented with 10% v/v fetal bovine serum (FBS), 1% v/v penicillin-streptomycin, 1% v/v L-glutamine and were maintained in a humidified atmosphere at 37°C and 5% CO_2_.

### Kynurenine measurement

The IDO activity was determined according to [24], with minor modifications: 200 μL of cell culture supernatants were added to 100 μL of 30% w/v trichloroacetic acid (TCA) and incubated for 30 min at 50°C to hydrolyze N-formylkynurenine to kynurenine. After centrifugation at 10,000 x g for 10 min, 100 μL of the supernatant were transferred into a 96-well plate, mixed with 100 μL of 2% w/v p-dimethylamino benzaldehyde in 99.8% v/v acetic acid, and incubated for 10 min at room temperature. Kynurenine was detected by measuring the absorbance at 490 nm, using a Synergy HT Multi-Detection Microplate Reader (BioTek, Winooski, VT). The absorbance of the culture medium alone was considered as a blank and was subtracted from the values obtained in the presence of the cells. The results were expressed as nmol kynurenine/mg cell proteins, according to a titration curve previously set. Kynurenine levels in the cell culture supernatants were measured in parallel by high pressure liquid chromatography (HPLC), as reported in [25], with minor modifications: 400 μL of the cell culture supernatants were added to 100 μL of 30% w/v TCA, incubated for 30 min at 50°C and centrifuged at 15,000 x g for 10 min. The clear supernatant was filtered through 0.45 μm PTFE filters (Alltech, Nicholasville, KY) and analyzed by an Agilent LC system (Palo Alto, CA), equipped with vacuum degasser (G1322A), quaternary pump (G1311A), manual-injector (Rheodyne, Cotati, CA) and multiple wavelength detector (G1365A) integrated in the HP1200 system. The data were acquired and processed with the Agilent ChemStation software. The injection volume was 20 μL. An Agilent Eclipse XDB-C18 column (125 mm × 4.0 mm, 5μm) was used for the analysis at 30°C. The chromatographic separation was carried out using the mobile phase consisting of 15 mmol/L acetate buffer (pH 4.0) and acetonitrile (90:10, v/v) at a flow rate of 0.8 mL/min. The eluate was monitored at 365 nm, referenced against a 700 nm wavelength. The calibration samples were prepared by adding kynurenine standards (Sigma Chemical Co.) in the concentration range of 0.50–50 μmol/L to the culture medium. The results were expressed as nmol kynurenine/mg cell proteins.

### qRT-PCR and PCR arrays

The total RNA was extracted and reverse-transcribed using the iScript cDNA Synthesis Kit (Bio-Rad Laboratories). The qRT-PCR was performed with the iTaq Universal SYBR Green Supermix (Bio-Rad Laboratories). The same cDNA preparation was used to quantify the gene of interest and the housekeeping gene *β-actin*. The primer sequences, designed with the qPrimerDepot software (http://primerdepot.nci.nih.gov/), were: for *IDO1*: 5’- CAGGCAGATGTTTAGCAATGA -3’; 5’- GATGAAGAAGTGGGCTTTGC -3’; for *β-actin*: 5’- GCTATCCAGGCTGTGCTATC-3’; 5’- TGTCACGCACGATTTCC-3’. The amount of *IDO1* mRNA was normalized versus the amount of *β-actin* mRNA, chosen as housekeeping gene, and was expressed as *IDO1*/*β-actin* ratio, using the Bio-Rad Software Gene Expression Quantitation (Bio-Rad Laboratories). The PCR arrays were performed on 1 μg cDNA, using the Human JAK/STAT Signaling Pathway RT² Profiler PCR Array and the Human IL-6/STAT3 Signaling Pathway Plus RT² Profiler PCR Array (Qiagen, Hilden, Germany), following the manufacturer’s instructions. The analysis of data was performed with the RT² Profiler PCR Array Data Analysis (Qiagen).

### Western blotting

The cells were rinsed with the lysis buffer (125 mmol/L Tris-HCl, 750 mmol/L NaCl, 1% v/v NP40, 10% v/v glycerol, 50 mmol/L MgCl_2_, 5 mmol/L EDTA, 25 mmol/L NaF, 1 mmol/L NaVO_4_, 10 μg/mL leupeptin, 10 μg/mL pepstatin, 10 μg/mL aprotinin, 1 mmol/L phenylmethylsulfonyl fluoride; pH 7.5), sonicated and centrifuged at 13,000 x g for 10 min at 4°C. 20 μg of proteins from cell lysates were subjected to Western blotting and probed with the following antibodies against: IDO1 (rabbit polyclonal, diluted 1:2,000, AG-25A-0029, Adipogen, San Diego, CA); IDO2 (mouse monoclonal, diluted 1:500, SAB3701447, Sigma Chemical Co.); TDO (rabbit polyclonal, diluted 1:1,000, SAB2102400, Sigma Chemical Co.); phospho(Tyr 1022/1023)-JAK1 (rabbit polyclonal, diluted 1:1,000, #3331, Cell Signaling Technology, Danvers, MA); JAK1 (rabbit polyclonal, diluted 1:1,000, #3344, Cell Signaling Technology); phospho(Tyr701)-STAT1 (rabbit polyclonal, diluted 1:1,000, #9167, Cell Signaling Technology); STAT1 (mouse monoclonal, diluted 1:1,000, clone 15H3, Thermo Scientific, Rockford, IL); phospho(Tyr705)-STAT3 (rabbit polyclonal, diluted 1:2,000, #9145, Cell Signaling Technology); STAT3 (mouse monoclonal, diluted 1:5,000, clone 9D8, Thermo Scientific); Pgp (rabbit polyclonal, diluted 1:250, sc-8313, Santa Cruz Biotechnology Inc.); MRP1 (mouse monoclonal, diluted 1:100, ab32574, Abcam, Cambridge, UK); MRP2 (mouse monoclonal, diluted 1:100, ab3373, Abcam); MRP3 (goat polyclonal, diluted 1:250, sc-5776, Santa Cruz Biotechnology Inc.); MRP4 (goat polyclonal, diluted 1:250, ab77184, Abcam); MRP5 (goat polyclonal, diluted 1:250, sc-5781, Santa Cruz Biotechnology Inc.); BCRP (rabbit polyclonal, diluted 1:500, sc-25882, Santa Cruz Biotechnology Inc.); β-tubulin (mouse monoclonal, diluted 1:500, sc-5274, Santa Cruz Biotechnology Inc.), followed by a secondary peroxidase-conjugated antibody (Bio-Rad Laboratories). The proteins were detected by enhanced chemiluminescence (Bio-Rad Laboratories). Nuclear extracts were prepared with the Nuclear Extract Kit (Active Motif, Rixensart, Belgium); 10 μg of nuclear proteins were resolved by SDS-PAGE and probed with the following antibodies against: PIAS1 (rabbit monoclonal, diluted 1:1,000, ab109388, Abcam); PIAS3 (rabbit polyclonal, diluted 1:1,000, ab22856, Abcam); phospho(Tyr701)-STAT1; STAT1; phospho(Tyr705)-STAT3; STAT3; TATA-binding protein (TBP; rabbit polyclonal, diluted 1.500, sc-273, Santa Cruz Biotechnology Inc.). To exclude any cytosolic contamination of nuclear extracts, we verified that β-tubulin was undetectable in nuclear samples (not shown).

### *In Vivo* Tumor Growth

1 x 10^5^ human A549, A549/dx, HT29, HT29/dx cells in 20 μL of culture medium, mixed with 20 μL of Cultrex BME (Trevigen, Gaithersburg, MD), were implanted subcutaneously in the right flank of 6–8 weeks old female nude BALB/c mice, housed under 12 h light/dark cycle, with food and drinking provided *ad libitum*. 1 x 10^5^ murine chemoresistant JC cells, syngeneic with BALB/c mice [26], were implanted in immunocompetent animals. In a second experimental set, when A549/dx, HT29/dx and JC tumors reached the volume of 100 mm^3^, the animals were randomized into two groups: “Control” group (treated with 100 μL of saline solution *per os*, 5 days/week for three weeks); “Brassinin” group (treated with 400 mg/kg of the IDO1 inhibitor 5-Br-brassinin *per os*, 5 days/week for three weeks), as described in [27]. In both experimental sets, the tumor growth was measured daily by caliper and was calculated according to the equation (LxW^2^)/2, where L = tumor length and W = tumor width. Mice were euthanized at day 21. The experimental procedures were approved by the Bioethics Committee (“Comitato Etico di Ateneo”) of the University of Torino, Italy.

### Cytokine production

The production of cytokines was measured in the cell culture supernatant using the following commercial kits: Human interleukin-6 (IL-6) Duo Set Development Kit (R&D Systems), Human interleukin-4 (IL-4) DuoSet Development Kit (R&D Systems), Human IL1-beta (IL-1β) platinum ELISA kit (eBioscience, San Diego, CA), Human interleukin-13 (IL-13) ELISA Development Kit (Peprotech, London, UK), Human soluble CD40 Ligand (sCD40L) ELISA Development Kit (Peprotech), Human tumor necrosis factor- α (TNF-α) DuoSet Development Kit (R&D Systems), Human IFN-γ DuoSet Development Kit (R&D Systems). Results were expressed as ng/mg cell proteins or pg/mg cell proteins, according to the calibration curve of each kit.

### Cell silencing

200,000 cells were transfected with 400 nmol/L of 19–25 nucleotide non targeting scrambled siRNAs (Control siRNA-A, Santa Cruz Biotechnology Inc.), with a STAT1- or STAT3-specific siRNAs pool (Santa Cruz Biotechnology Inc.), according to the manufacturer’s instructions. To verify the silencing efficacy, the cells were lysed and checked for the expression of STAT1 and STAT3 by Western blotting, as described above.

### Immunological assays

1 x 10^6^/mL of human peripheral blood mononuclear cells (PBMC), isolated from buffy coats of healthy donors (Blood Bank, A.O.U. Città della Salute e della Scienza di Torino Hospital, Torino, Italy) by centrifugation on Ficoll-Hypaque density gradient, were treated with anti-CD3 (OKT3, BioLegend, San Diego, CA) and anti-CD28 (BioLegend) antibodies, to induce the specific proliferation of T-lymphocytes, and co-cultured with target cells (previously irradiated with 30 Gy for 15 min) for 72 h at an effector/target ratio of 10:1. The expansion of T-lymphocytes, the only PBMC population able to proliferate in these experimental conditions, was assessed by adding 1 μCi of [^3^H]thymidine (PerkinElmer, Waltham, MA) 18 h before the end of the co-cultures, then harvesting the plates and counting the radioactivity. To analyze the lymphocyte phenotype after the incubation with tumor cells, the cells were harvested, washed and re-suspended in phosphate buffer saline (PBS) containing 5% v/v FBS. A 3- and 4-color flow cytometry analysis was performed with the appropriate combinations of fluorescein isothiocyanate-, r-phycoerythrin-, tricolor-, peridinin chlorophyll protein complex—or allophycocyanin-conjugated antibodies for CD3, CD4, CD8, CD25 (all from Miltenyi Biotech, Bergisch Gladbach, Germany), and CD127 (BioLegend). Isotype controls were run for each sample. The samples were read with a FACS Calibur flow cytometer equipped with a CELLQuestPro software (Becton Dickinson). The use of PBMC from healthy donors and the experimental protocols were approved by the Bioethics Committee (“Comitato Etico Interaziendale”) of the A.O.U. Città della Salute e della Scienza di Torino Hospital, Torino, Italy.

### Cell viability assay

The neutral red staining was performed to measure the cell viability, as previously detailed [28]. The absorbance at 540 nm was read using a Synergy HT Multi-Detection Microplate Reader. The absorbance of untreated cells was considered as 100% viability; the results were expressed as percentage of viable cells versus untreated cells.

### Nitrite measurement

The level of nitrite, a stable derivative of NO, in the cell culture supernatants was measured by the Griess method [29]. The results were expressed as nmol nitrite/mg cell proteins.

### Iron measurement

100 x 10^6^ cells were washed with PBS, detached with trypsin/EDTA, centrifuged at 12,000 x g for 2 min, re-suspended in 1 mL PBS and sonicated. The intracellular iron was measured using a AAnalyst 200 Atomic Absorption Spectrometer (PerkinElmer). The results were expressed as ng iron/ml cell suspension.

### Statistical analysis

All data in text and figures are provided as means ± SD. The results were analyzed by a one-way analysis of variance (ANOVA). A *p* < 0.05 was considered significant.

## Results

### Kynurenine synthesis is higher in multidrug resistant cells and is modulated by 5-Br-brassinin, methyl-DL-tryptophan and interferon-γ

**Fig 1:**
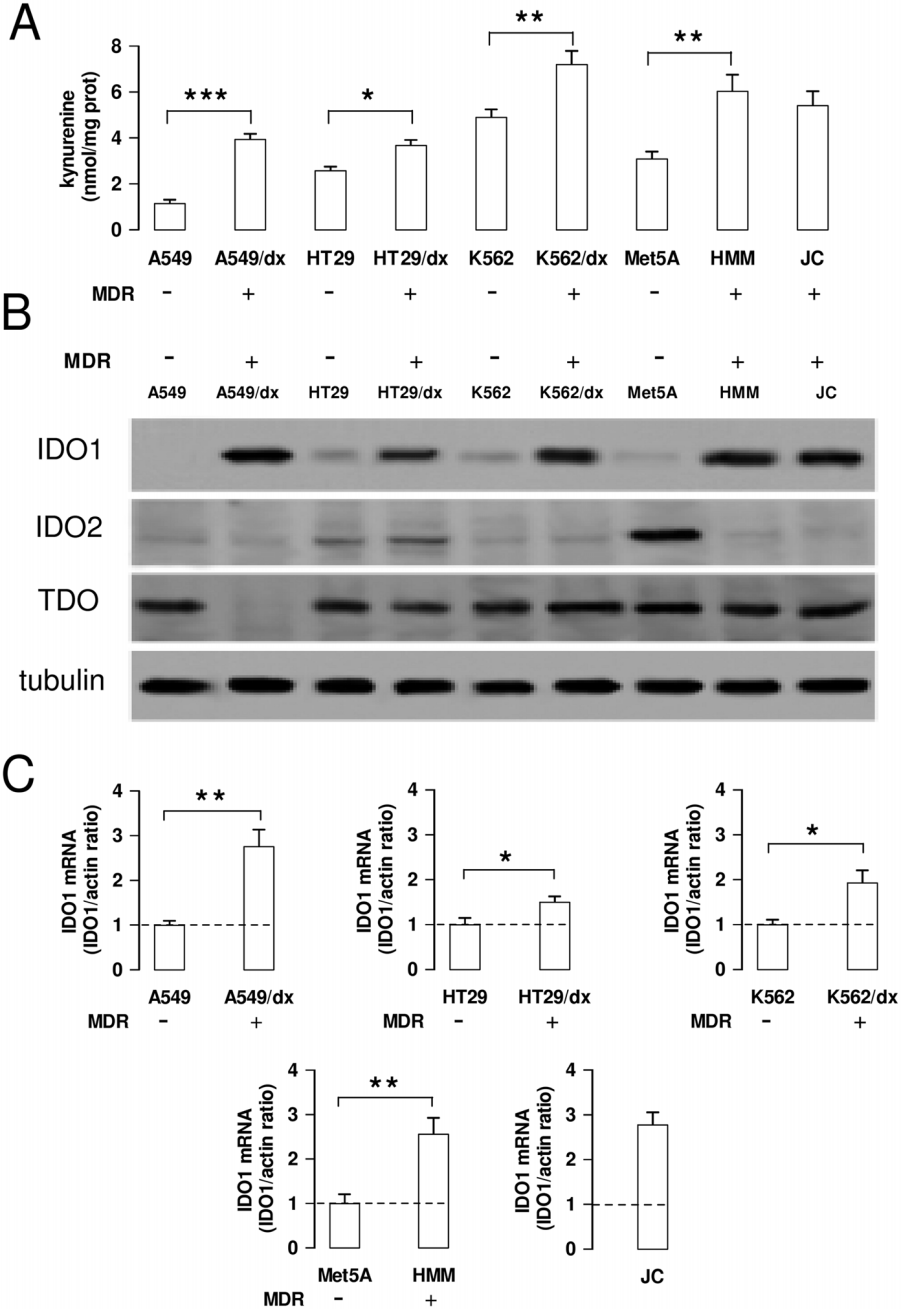
Kynurenine production and IDO1 expression in chemosensitive and multidrug resistant cells. Human chemosensitive lung cancer A549 cells and chemoresistant A549/dx cells, human chemosensitive colon cancer HT29 cells and chemoresistant HT29/dx cells, human chemosensitive chronic myelogenous leukemia K562 cells and chemoresistant K562/dx cells, human chemosensitive mesothelial Met5A cells and human chemoresistant malignant mesothelioma HMM cells, murine chemoresistant mammary JC cells were subjected to the following investigations. **A**. The kynurenine levels in the cell culture supernatants were measured spectrophotometrically. Data are presented as means ± SD (n = 4). * p < 0.05, ** p < 0.01, *** p < 0.001: chemoresistant cells (MDR-positive) versus the corresponding chemosensitive (MDR-negative) cells. **B**. Western blot analysis of IDO1, IDO2 and TDO expression. The β-tubulin expression was used as control of equal protein loading. The figure is representative of 3 experiments with similar results. **C**. The expression level of *IDO1* mRNA was measured by qRT-PCR. Data are presented as means ± SD (n = 4). * p < 0.01, ** p < 0.002: chemoresistant cells (MDR-positive) versus the corresponding chemosensitive (MDR-negative) cells.

We first analyzed the kynurenine production in a panel of chemosensitive and multidrug resistant cancer cells, showing a different pattern of ABC transporters (S1 Fig): HT29, A549, K562, Met5A were human chemosensitive cells; HT29/dx, A549/dx and K562/dx were models of acquired MDR; HMM and JC were human and murine constitutively chemoresistant cells, respectively. The multidrug resistant cell lines had higher kynurenine levels—detected by HPLC (S2 Fig) and spectrophotometric assay (Fig 1A)—and increased levels of IDO1 protein compared to chemosensitive cells (Fig 1B). IDO2 was detected at variable amounts in chemosensitive and chemoresistant cells; TDO was detected in all the cell lines analyzed, except in A549/dx cells (Fig 1B). *IDO1* mRNA resulted also higher in multidrug resistant cells than in chemosensitive ones (Fig 1C).

**Fig 2:**
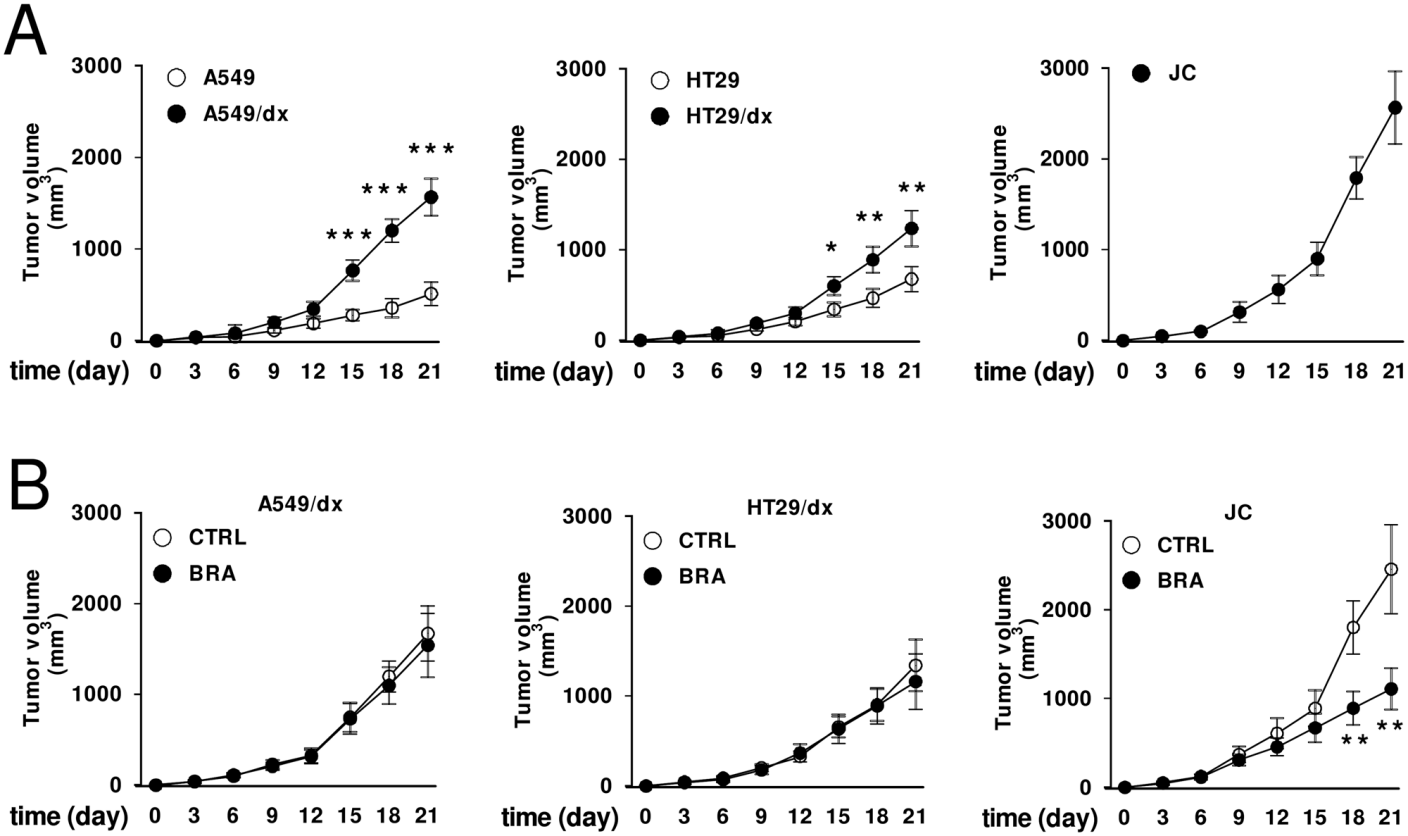
Effects of IDO1 inhibition on the growth of multidrug resistant tumors. **A**. 1 x 10^5^ human A549, A549/dx, HT29, HT29/dx cells were implanted subcutaneously in 6–8 weeks old female nude BALB/c mice, 1 x 10^5^ murine JC cells were implanted in immunocompetent BALB/c mice. Tumor growth was monitored daily by caliper measurement. Data are presented as means ± SD of 10 mice/group. * p < 0.02, ** p < 0.005, *** p < 0.001: A549/dx or HT29/dx cells versus A549 or HT29 cells, at the corresponding time points. **B**. Animals bearing A549/dx-, HT29/dx-, JC-tumors were randomized into two groups when tumors reached the volume of 100 mm^3^: “Control” group (treated with 100 μL of saline solution *per os*, 5 days/week for three weeks; CTRL); “Brassinin” group (treated with 400 mg/kg of the IDO1 inhibitor 5-Br-brassinin *per os*, 5 days/week for three weeks; BRA). Tumor growth was monitored daily by caliper measurement. Data are presented as means ± SD of 6 mice/group. ** p < 0.005: BRA-group versus CTRL-group, at the corresponding time points.

Human chemoresistant A549/dx cells and HT29/dx cells grew faster than the chemosensitive A549 and HT29 cells implanted in nude BALB/c mice (Fig 2A). The murine chemoresistant JC cells had the highest rate of growth (Fig 2A). Interestingly, the IDO1 inhibitor 5-Br-brassinin [27] did not inhibit the growth of A549/dx and HT29/dx tumors, implanted in immunodeficient mice, but it significantly reduced the progression of JC tumors, implanted in immunocompetent animals (Fig 2B). These data suggest that IDO activity may support the fast growth of chemoresistant tumors in immunocompetent hosts.

We next investigated the reason of the difference in kynurenine production between chemosensitive and chemoresistant cells. For sake of simplicity, we focused on the A549 and A549/dx cells as models of chemosensitive and multidrug resistant cells, respectively, since in these cells the difference in kynurenine levels and IDO1 expression was very evident. Since the HPLC measurement and the spectrophotometric assay gave superimposable results for A549 and A549/dx cells (S2 Fig and Fig 1A), we used the latter assay as a reliable method to evaluate the differences in the kynurenine levels between these two cell lines.

We analyzed whether kynurenine levels varied differently in response to chemotherapeutic drugs—to which multidrug resistant cells are insensitive—, to IDO1 activators, such as NO, iron and IFN-γ, and to IDO1 inhibitors, such as 5-Br-brassinin and methyl-DL-tryptophan [30].

Doxorubicin, cisplatin, gemcitabine and mitoxantrone, used at concentrations that reduced to 50% the viability of sensitive A549 cells without affecting the viability of resistant A549/dx cells (S3A Fig), did not change the kynurenine levels, which remained significantly higher in A549/dx cells than in A549 cells, either in the absence or in the presence of the chemotherapeutic agents (S3B Fig). Similarly, the NO donors S-nitrosoglutathione and S-nitroso-N-acetylpenicillamine, which increased the levels of NO in chemosensitive and multidrug resistant cells (S4A Fig), did not modify the kynurenine synthesis compared to untreated cells in both cell populations (S4B Fig). To modulate the intracellular iron, we treated A549 and A549/dx cells with the cell permeable iron-releasing compound FeNTA and with the iron chelator desferroxamine, which respectively increased and decreased the amount of cell iron (S5A Fig): again, neither FeNTA nor desferroxamine varied the kynurenine production (S5B Fig).

**Fig 3:**
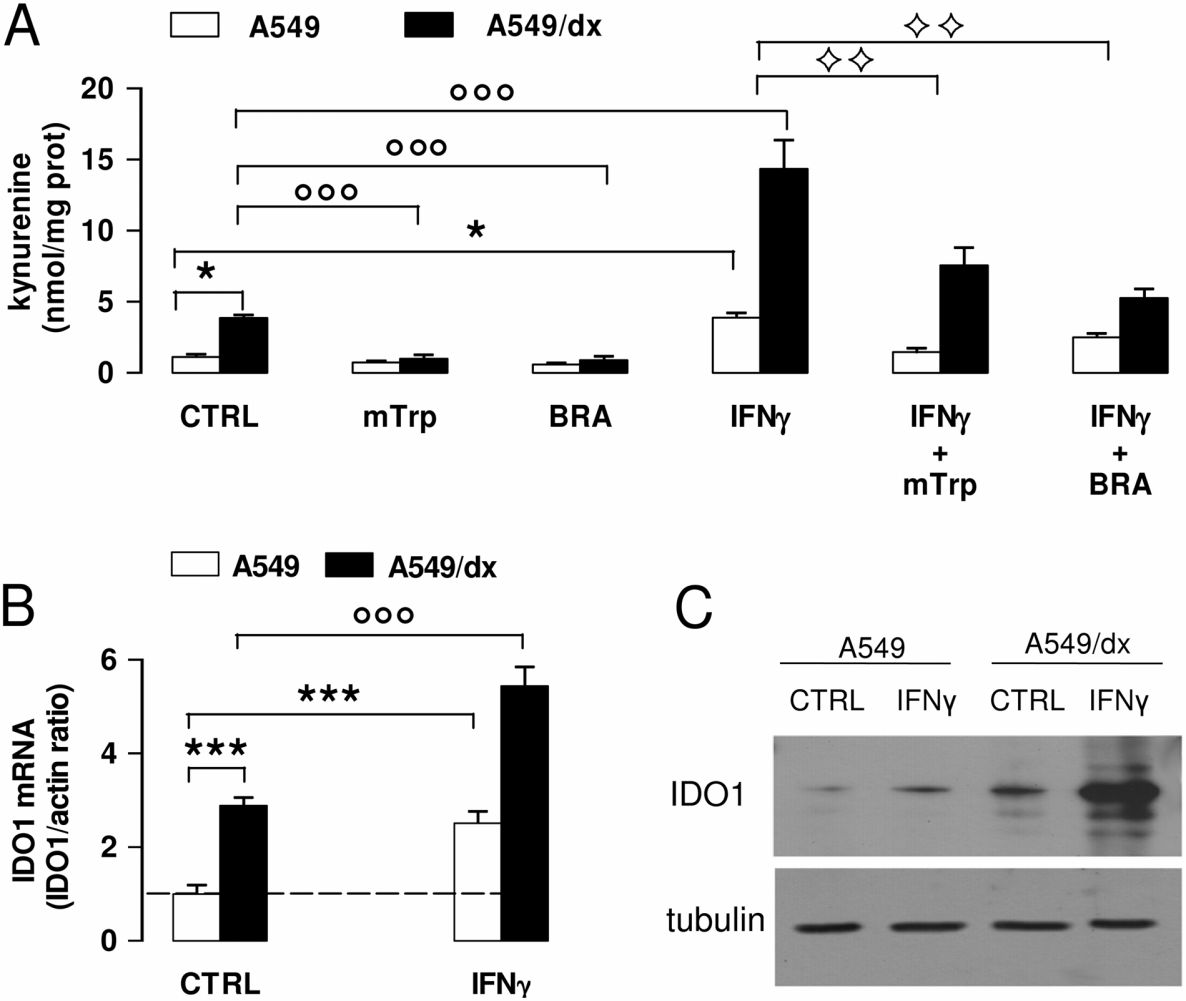
Effects of IFN-γ on kynurenine synthesis and IDO1 expression in chemosensitive and multidrug resistant cells. A549 and A549/dx cells were incubated for 48 h in fresh medium (CTRL) or in medium containing the IDO1 inhibitors methyl-DL-tryptophan (1 mmol/L, mTrp) or 5-Br-brassinin (100 μmol/L, BRA), and the IDO1 inducer IFN-γ (100 ng/mL, IFNγ), alone or in combination. **A**. The kynurenine levels in the cell culture supernatants were measured spectrophotometrically. Data are presented as means ± SD (n = 4). * p < 0.01: versus A549 CTRL cells; °°° p < 0.001: versus A549/dx CTRL; ^◊◊^ p < 0.005: IFN-γ + mTrp-treated, IFN-γ + BRA-treated A549 and A549/dx cells versus the corresponding cells treated with IFN-γ alone. **B**. The expression level of *IDO1* mRNA was measured by qRT-PCR. Data are presented as means ± SD (n = 4). *** p < 0.001: versus A549 CTRL cells; °°° p < 0.001: versus A549/dx CTRL cells. **C**. Western blot analysis of IDO1 expression. The β-tubulin expression was used as control of equal protein loading. The figure is representative of 3 experiments with similar results.

IFN-γ increased the kynurenine levels in both chemosensitive and multidrug resistant cells, but the extent of such increase was greater in A549/dx cells (Fig 3A). The effect of IFN-γ, which was reduced by the inhibitors methyl-DL-tryptophan and 5-Br-brassinin (Fig 3A), was associated to the increase of *IDO1* mRNA (Fig 3B) and protein (Fig 3C). Also in this case, the increase elicited by IFN-γ was more pronounced in A549/dx than in A549 cells (Fig 3B and 3C), suggesting that the multidrug resistant cells were more responsive to the cytokine. Similar effects of IFN-γ, methyl-DL-tryptophan and 5-Br-brassinin were detected in HT29 and HT29/dx cells, K562 and K562/dx cells, Met5A and HMM cells (data not shown).

### Multidrug resistant cells have a higher activity of JAK/STAT signaling and an increased autocrine production of STAT3-dependent cytokines than chemosensitive cells

**Fig 4:**
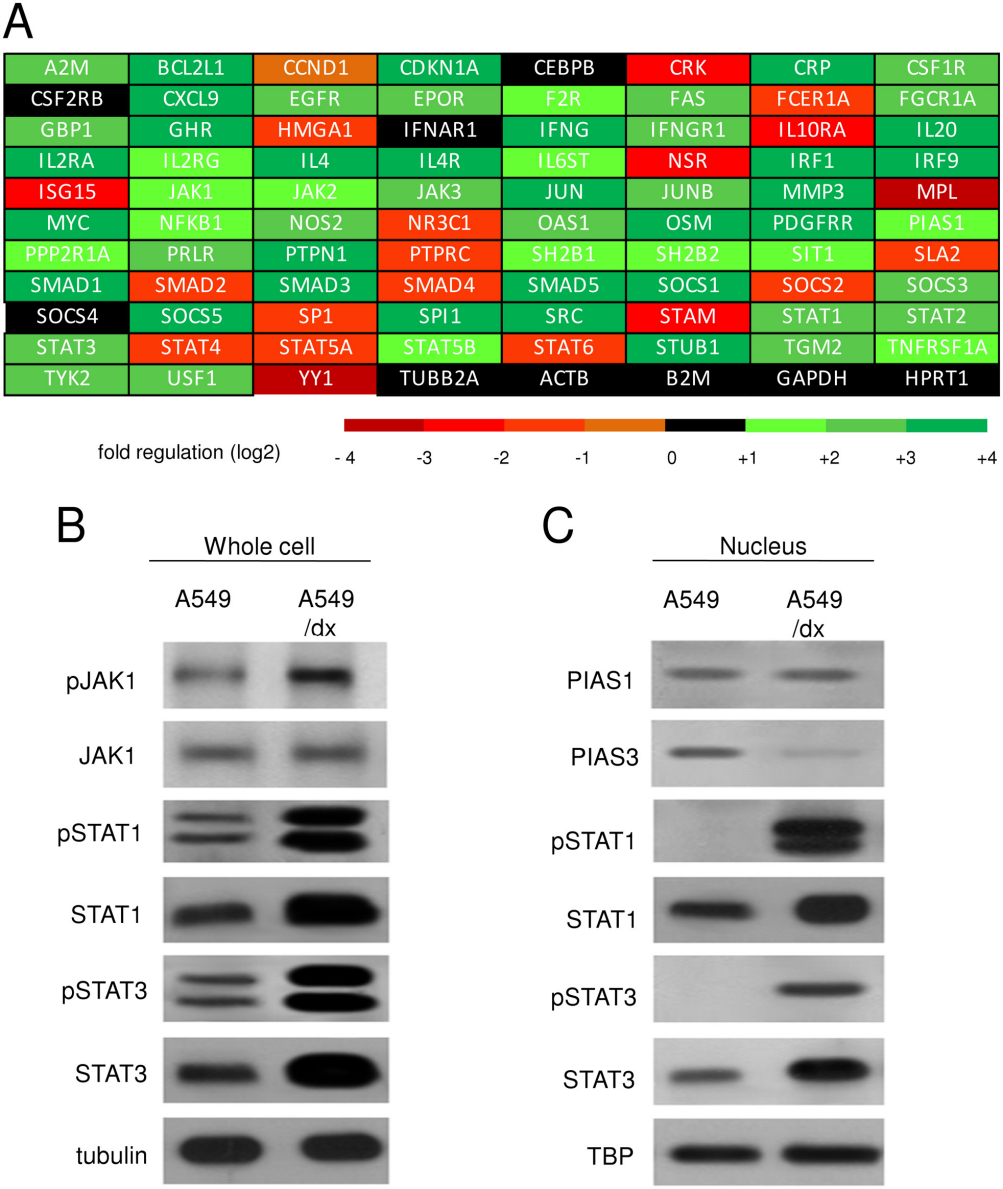
Multidrug resistant cells have a higher activity of JAK/STAT signaling than chemosensitive cells. A. The cDNA from A549 and A549/dx cells was analyzed by a PCR array specific for JAK/STAT signaling, as reported under Materials and methods. The fold regulation of the 83 genes analyzed, expressed in logarithmic scale, is represented in a colorimetric scale. The figure is the mean of 4 experiments. B. The cells were lysed and subjected to the Western blot analysis for phospho(Tyr 1022/1023)-JAK1, JAK1, phospho(Tyr701)-STAT1, STAT1, phospho(Tyr705)-STAT3, STAT3. The β-tubulin expression was used as control of equal protein loading. The figure is representative of 3 experiments with similar results. C. The expression of PIAS1, PIAS3, phospho(Tyr701)-STAT1, STAT1, phospho(Tyr705)-STAT3, STAT3 in nuclear extracts was measured by Western blotting. The TBP expression was used as control of equal protein loading. The figure is representative of 3 experiments with similar results.

Since IFN-γ activates the JAK/STAT1-3 signaling [31], and STAT1 and STAT3 are potent transcriptional activators of the *IDO1* gene in most mammalian cells [32, 33], we analyzed the expression levels of key genes of JAK/STAT pathway by a high-throughput PCR screening. As shown in S1 Table and Fig 4A, A549/dx cells did not differ from A549 cells for the expression of *JAK1-2-3*, but exhibited higher expression of *STAT1* and *STAT3*. In keeping with this trend, classical STAT1- and STAT3-target genes (*A2M*, *BCL2L1*, *CDKN1A*, *CRP*, *CXCL9*, *FAS*, *IRF1*, *JUNB*, *MMP3*, *MYC*, *NOS2*, *SOCS1*) were up-regulated in the multidrug resistant cells (S1 Table). In Western blotting validation, we found similar levels of total JAK1 protein in whole cell lysates of A549 and A549/dx cells, but higher levels of the active tyrosine-phosphorylated JAK1 in the multidrug resistant cells (Fig 4B). The amounts of STAT1, phospho(Tyr701)-STAT1, STAT3, phospho(Tyr705)-STAT3 were also higher in the chemoresistant cells (Fig 4B). The mRNA level of the STAT1 inhibitor PIAS1 was not significantly different between A549 and A549/dx cells (S1 Table and Fig 4A), and the level of PIAS1 protein was the same in the nuclear extracts (Fig 4C). By contrast, the nuclear amount of the STAT3 inhibitor PIAS3 was lower in A549/dx cells (Fig 4C); this was in line with the lower level of *PIAS3* mRNA (S2 Table). Interestingly, STAT1, phospho(Tyr701)-STAT1, STAT3, phospho(Tyr705)-STAT3 were all more translocated into the nucleus of multidrug resistant cells (Fig 4C). This pattern, which is likely due to the higher amount and phosphorylation of STAT1/STAT3 and to the lower expression of PIAS3, led us to hypothesize that STAT1- and, in particular, STAT3-target genes should be up-regulated in the multidrug resistant cells.

**Fig 5:**
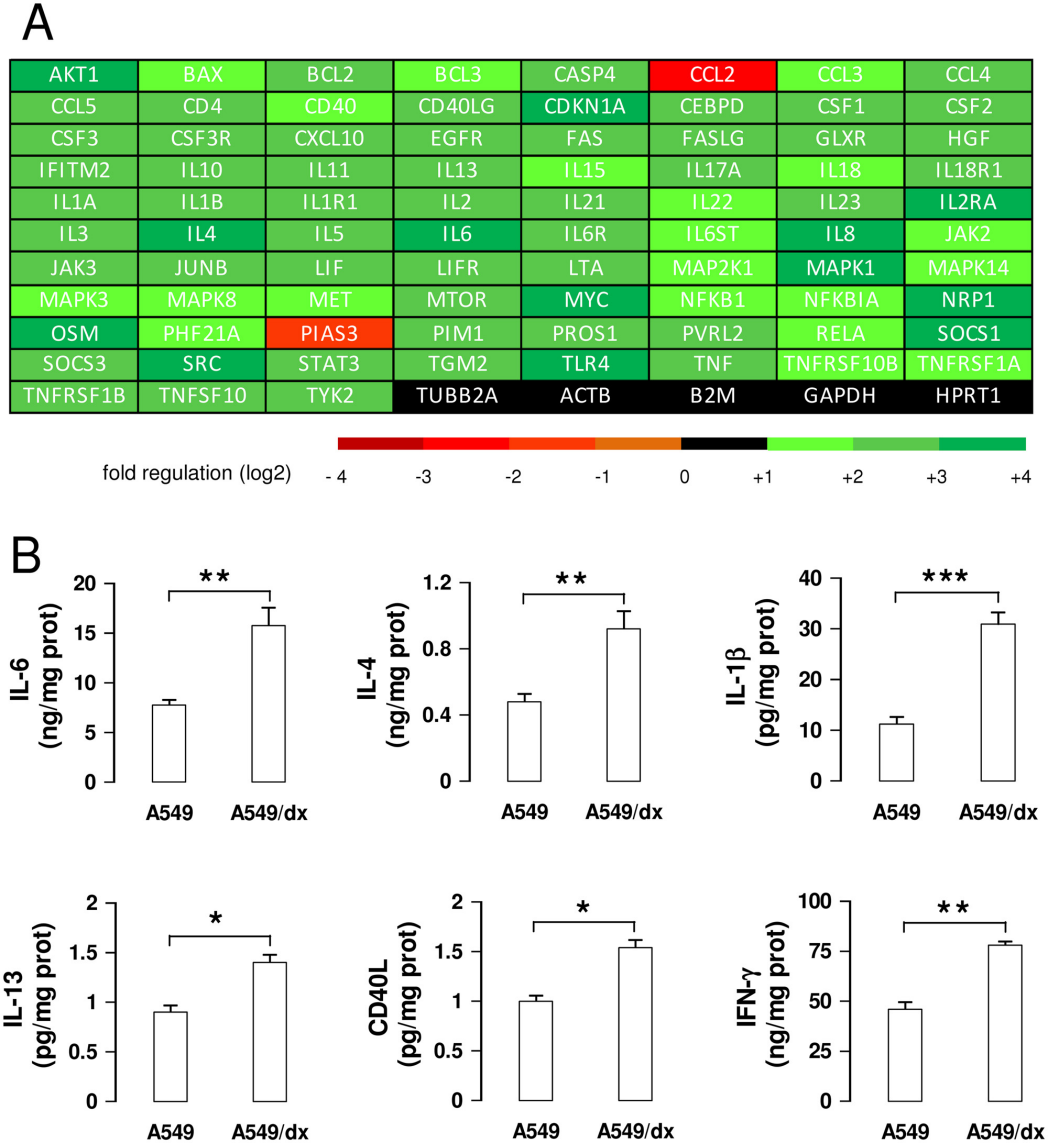
Multidrug resistant cells have a higher activity of IL-6/STAT3 signaling than chemosensitive cells. A. The cDNA from A549 and A549/dx cells was analyzed by a PCR array specific for IL-6/STAT3 signaling, as reported under Materials and methods. The fold regulation of the 83 genes analyzed, expressed in logarithmic scale, was represented in a colorimetric scale. The figure is the mean of 4 experiments. B. The levels of IL-6, IL-4, IL-1β, IL-13, CD40L, IFN-γ were measured in the cell culture supernatants by specific ELISAs. Data are presented as means ± SD (n = 3). * p < 0.02, ** p < 0.005, *** p < 0.001: A549/dx cells versus A549 cells.

Indeed a global up-regulation of the STAT3-dependent genes was detected in A549/dx cells (S2 Table and Fig 5A). Of note, the mRNAs of the STAT3-target cytokines IL-6, IL-4, IL-1β, IL-13, CD40L, TNF-α—that are IDO1 inducers [34–38]—were up-regulated more than two-fold in A549/dx cells (S2 Table and Fig 5A). The ELISA assays confirmed the higher autocrine production of these cytokines in the multidrug resistant cells (Fig 5B), except in the case of TNF-α, whose level was below the detection limit of the kit (data not shown). Also the IFN-γ mRNA (S1 Table and Fig 4A) and protein (Fig 5B) were higher in A549/dx cells, increasing the number of the IDO-inducer cytokines produced by these cells.

### STAT1/STAT3 silencing decreases the up-regulation of IDO1 and the kynurenine-induced immunosuppression in multidrug resistant cells

**Fig 6:**
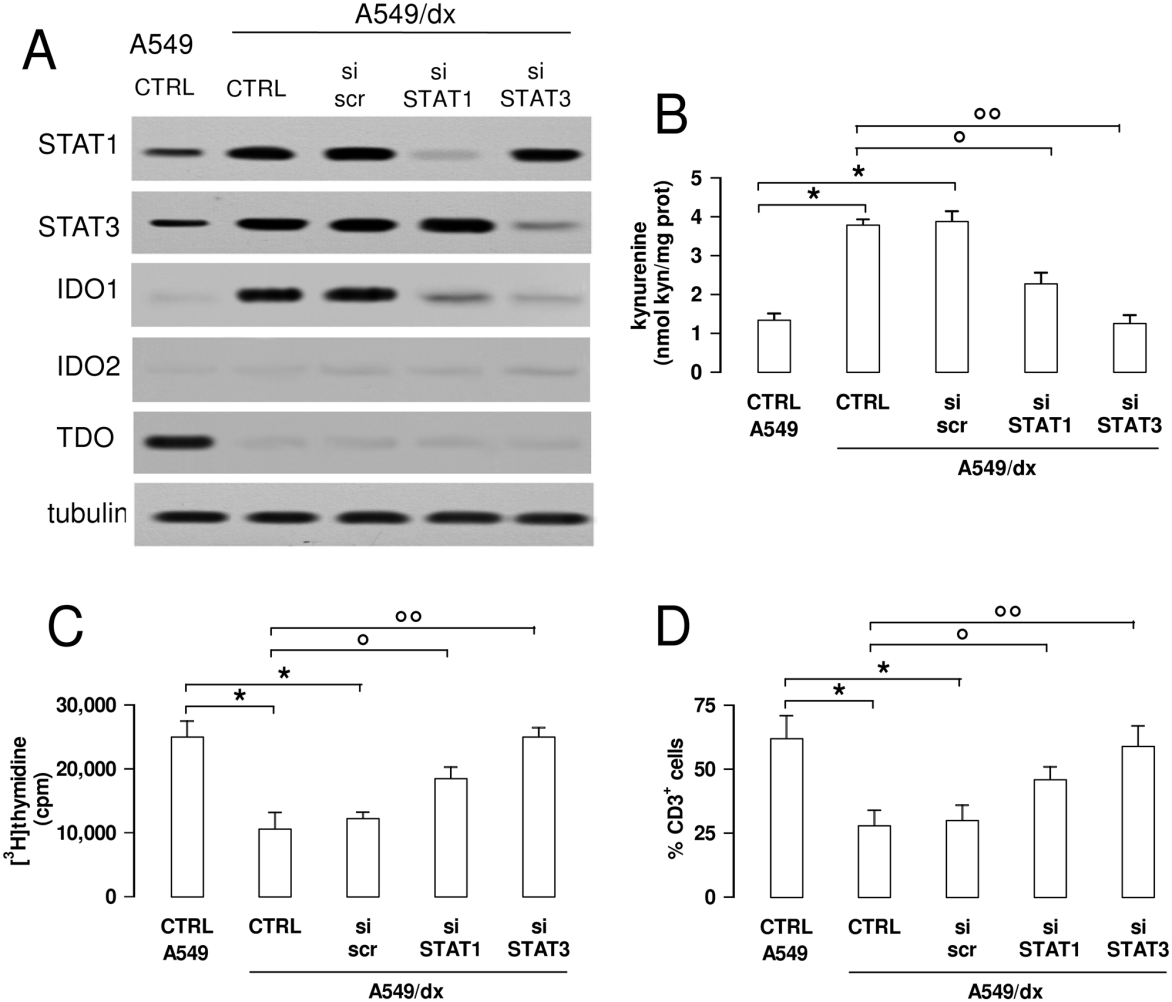
The inhibition of the STAT1/STAT3 signaling reverses the kynurenine-dependent immunosuppression in multidrug resistant cells. A549/dx cells were grown for 48 h in fresh medium (CTRL), treated with a non-targeting scrambled siRNA (scr) or with a specific siRNAs pool targeting STAT1 or STAT3, respectively (si STAT1, si STAT3). Untreated chemosensitive A549 cells were used as control. **A**. The expression of STAT1, STAT3, IDO1, IDO2 and TDO was measured in whole cell lysates by Western blotting, 48 h after the transfection. The β-tubulin expression was used as control of equal protein loading. The figure is representative of 3 experiments with similar results. **B**. The kynurenine levels in the cell culture supernatants were measured spectrophotometrically. Data are presented as means ± SD (n = 4). * p < 0.01: versus A549 CTRL; ° p < 0.005, °° p < 0.001: versus A549/dx CTRL. **C**. The proliferation of activated T-lymphocytes collected from PBMC after a 72 h co-incubation with A549 and A549/dx cells was measured with the [^3^H]thymidine assay. In the presence of anti-CD3 and anti-CD28 stimulatory antibodies without tumor cells (positive control), the [^3^H]thymidine incorporation was 28,926 ± 1,426 cpm; in the presence of RPMI medium alone (negative control), the [^3^H]thymidine incorporation was 4,312 ± 529 cpm. Data are presented as means ± SD (n = 6). * p < 0.05: versus A549 CTRL; ° p < 0.01, °° p < 0.005: versus A549/dx CTRL. **D**. The percentage of CD3^+^ T-lymphocytes collected from PBMC, co-incubated with tumor cells as reported in **C**, was measured by flow cytometry. Data are presented as means ± SD (n = 6). * p < 0.01: versus A549 CTRL; ° p < 0.01, °° p < 0.002: versus A549/dx CTRL.

To validate the functional role of STAT1 and STAT3 as IDO1 inducers in multidrug resistant cells, we produced A549/dx clones transiently silenced for each protein (Fig 6A). STAT3- and, to a lower extent, STAT1-silenced cells showed lower levels of IDO1 protein, without changes in IDO2 and TDO amount (Fig 6A). Silenced cells also had a reduced kynurenine synthesis, which in STAT3-silenced A549/dx cells was superimposable to the one of A549 cells (Fig 6B).

In keeping with the different expression of IDO1 and kynurenine levels, the chemosensitive A549 cells stimulated the proliferation of T-lymphocytes more than the multidrug resistant A549/dx cells; the silencing of STAT1 and, in particular, of STAT3 in A549/dx cells restored the proliferation of T-cells to the same level of A549 cells (Fig 6C), which produced less kynurenine. In line with these results, the percentages of CD3^+^ cells recovered at the end of the co-incubation of PBMC with tumor cells was lower with A549/dx cells than with A549 cells, but the silencing of STAT1 and STAT3 significantly increased it (Fig 6D). No changes occurred in the proportion of CD4^+^ T-helper cells, CD8^+^ T-cytotoxic cells (S6A Fig) and Treg cells (S6B Fig), a typical subpopulation of T lymphocytes involved in immunotolerance and immunosuppression [39]. The same increase in T-lymphocyte proliferation (S7A Fig) and CD3^+^ expansion (S7B Fig) obtained after STAT-silencing was produced by treating A549/dx cells with the IDO1 inhibitor 5-Br-brassinin. These data suggest that the high kynurenine levels can be responsible for the reduced expansion of CD3^+^ T-lymphocytes induced by multidrug resistant cells.

## Discussion

In this work we report for the first time that kynurenine production was higher in multidrug resistant than in chemosensitive cancer cells, independently from the type of chemoresistance (i.e. chemotherapy-induced or constitutive MDR) and the type of tumor. According to the expression pattern of IDO1, IDO2 and TDO in chemosensitive and chemoresistant cell lines, the higher synthesis of kynurenine in resistant cells was mainly due to the higher amount of IDO1.

It has been recently reported that a high intratumor amount of IDO1 favors the growth of lung cancers [22]. This observation is in keeping with our *in vivo* experiments, where the chemoresistant tumors expressing high levels of IDO1 grew faster than the chemosensitive counterparts with low levels of the enzyme. The pharmacological inhibition of IDO1 with 5-Br-brassinin significantly reduced the growth of chemoresistant tumors in immunocompetent animals, but not in immunodeficient mice. This result, which was in agreement with previous observations [27], led to the hypothesis that the antitumor effects of IDO1 inhibition require an active immune system and are mediated by the reduction of immunosuppressive cells and/or by the restoration of immune cells active against multidrug resistant tumors.

To clarify the molecular mechanisms at the basis of IDO1 overexpression in multidrug resistant cells, we focused on the model of non small cell lung cancer A549 and A549/dx cells, where the difference of IDO1 expression between sensitive and resistant cells was particularly pronounced. A recent study demonstrated that the serum kynurenine/tryptophan ratio is higher in patients affected by non small cell lung cancer compared with healthy controls and that such difference was further increased by radio-chemotherapy [40]. Differently from what it was observed in patients, we did not detect any increase of kynurenine production in cells exposed to one dose of chemotherapeutic drugs. We cannot exclude however that repeated exposures to chemotherapeutic agents, as it occurs in patients, might further increase the kynurenine synthesis also in our *in vitro* model.

The most striking difference between chemosensitive and multidrug resistant cells was the constitutive activation of the JAK/STAT axis in resistant cells. Although the expression of JAK1, a documented activator of STAT1 and STAT3 [31, 41], was not different, the multidrug resistant cells showed a higher basal amount of STAT1 and STAT3, and of the activated tyrosine-phosphorylated forms of JAK1, STAT1 and STAT3. The phosphorylation on tyrosine of STATs is necessary for their homodimerization and translocation into the nucleus [31]: indeed nuclear phospho(Tyr701)-STAT1 and phospho(Tyr705)-STAT3, which were undetectable in chemosensitive cells, were abundant in multidrug resistant ones. These results suggest that both the increased transcription of STAT1 and STAT3, as revealed by the PCR array analysis, and the increased activity of the JAK/STAT axis, as indicated by the higher phosphorylation of STAT1/3, contribute to the increased activation of STATs in multidrug resistant cells and to the transcriptional activation of *IDO1* gene. Whereas the expression of the STAT1 inhibitor PIAS1 was equal in A549 and A549/dx cells, the amount of the STAT3 inhibitor PIAS3 was lower in A549/dx cells. According to this pattern, STAT3 more than STAT1 should have the higher transcriptional activity in our multidrug resistant cells. Indeed we found several STAT3-target genes significantly up-regulated in A549/dx cells; moreover, the selective silencing of STAT1 or STAT3 demonstrated that both factors were involved in *IDO1* transcription in multidrug resistant cells, but STAT3 had a slightly prominent role in this process.

The STAT1 and STAT3 activity is promoted by IFN-γ [31], a known IDO1 inducer [10]. In A549/dx cells the exogenous IFN-γ elicited a strong induction of IDO1, more evident than in A549 cells. This result suggests that the IFN-γ-dependent signaling was more promptly activated in multidrug resistant than in chemosensitive cells: this may be explained by the higher activity of JAK/STAT axis in A549/dx cells. Interestingly, we detected an autocrine production of IFN-γ, higher in A549/dx than in A549 cells: although the amount of IFN-γ was significantly lower than the one produced by activated immune system cells [42], it might be sufficient to induce the transcription of *IDO1* in multidrug resistant cells, owing to their constitutively activated JAK/STAT axis. Therefore, an autocrine IFN-γ/JAK/STAT loop may be the driving force for the induction of IDO1 in A549/dx cells. The maximal induction of IDO1, however, is often produced by the cooperation of IFN-γ with other cytokines [15]: IL-6, IL-4, IL-1β, IL-13, TNF-α and CD40L are documented inducers of IDO1, alone or together with IFN-γ [34–38]. According to the PCR array analysis, these cytokines were at least two-fold up-regulated in A549/dx cells, where they may contribute to the transcription of IDO. All the above-mentioned cytokines are STAT3-target genes; moreover, IL-6, IL-4 and IL-13 activate the JAK1/STAT3 axis with a feed-forward mechanism [43–45]. We might speculate that the higher basal production of these cytokines feeds multiple autocrine “cytokine/JAK/STAT3” loops, which sustain the transcription of *IDO1* in multidrug resistant cells.

The expression of IDO1 in non small cell lung cancer cells has been already related to the constitutive activation of the IL-6/STAT3 signaling: kynurenine activates the transcription factor aryl hydrocarbon receptor, that in turn increases the autocrine synthesis of IL-6 and the subsequent activation of STAT3 [34]. Although in this work it has not been investigated whether the cells were chemosensitive or chemoresistant, it is conceivable that a similar loop works in A549/dx cells. On the other hand, with a feedback mechanism, IL-6 increases the STAT3-signaling inhibitor protein SOCS3, which induces the proteasomal degradation of IDO1 [46]. *SOCS3* mRNA was indeed up-regulated in A549/dx cells: this up-regulation may perhaps buffer the effect of IL-6 on STAT3 activity and *IDO1* transcription, limiting the maximal activation of the enzyme. We believe that the concomitant production of multiple cytokines, more than a single cytokine, contributes to the activation of STAT3 and IDO1 in A549/dx cells.

To the best of our knowledge, only indirect evidences have correlated the activity of STAT3 and IDO1 to the chemoresistance. For instance, the disruption of the STAT3 signaling has reduced tumor proliferation and angiogenesis [47], and has sensitized resistant breast cancer cells to doxorubicin [48]. A high production of kynurenine has been associated with resistance to olaparib, a poly(ADP-ribose) polymerase inhibitor, gamma radiations and cisplatin [49]. On the other hand, the IDO1 inhibitor methyl-DL-tryptophan has increased the efficacy of paclitaxel in endometrial cancer xenografts [50], suggesting that the inhibition of IDO1 may improve the chemosensitivity. Our assays suggest an additional benefit of inhibiting the STAT3/IDO1 axis in resistant cells, i.e. the restoration of a significant expansion of T-lymphocytes that would be otherwise suppressed in the presence of multidrug resistant tumor cells.

Our work is the first demonstration that multidrug resistant cells have a higher endogenous production of kynurenine, sustained by the constitutive activation of a “cytokine/JAK/STAT3/IDO1” axis. Such phenotype is paralleled by the reduced expansion of the global population of T-lymphocytes, a feature that might favor the immune-evasion of multidrug resistant cells. Some pharmacological inhibitors of IDO1 [51] and the vaccination against IDO1 [52] are under evaluation in immunotherapy protocols to contrast the development of tumors refractory to the conventional therapies. In our multidrug resistant cells IDO1, although over-expressed, was sensitive to classical inhibitors such as methyl-DL-tryptophan and 5-Br-brassinin. This observation may be useful in a translational perspective and may represent a strong indication for the inclusion of IDO1 inhibitors in chemo-immunotherapy protocols against chemoresistant cancers, in order to limit the immunosuppressive attitude of these tumors.

## Supporting Information

S1 Fig Expression of ABC transporters in chemosensitive and multidrug resistant cells. Human chemosensitive lung cancer A549 cells and chemoresistant A549/dx cells, human chemosensitive colon cancer HT29 cells and chemoresistant HT29/dx cells, human chemosensitive chronic myelogenous leukemia K562 cells and chemoresistant K562/dx cells, human chemosensitive mesothelial Met5A cells and human chemoresistant malignant mesothelioma HMM cells, murine chemoresistant mammary JC cells were lysed and subjected to the Western blot analysis for Pgp, MRP1, MRP2, MRP3, MRP4, MRP5, BCRP. The β-tubulin expression was used as control of equal protein loading. The figure is representative of 3 experiments with similar results. MDR-: chemosensitive cell lines; MDR +: chemoresistant cell lines.
(TIF)

S2 Fig HPLC measurement of kynurenine levels in chemosensitive and multidrug resistant cells. The amount of kynurenine was measured by HPLC in the culture supernatants of human chemosensitive lung cancer A549 cells and chemoresistant A549/dx cells, human chemosensitive colon cancer HT29 cells and chemoresistant HT29/dx cells, human chemosensitive chronic myelogenous leukemia K562 cells and chemoresistant K562/dx cells, human chemosensitive mesothelial Met5A cells and human chemoresistant malignant mesothelioma HMM cells, murine chemoresistant mammary JC cells. Data are presented as means ± SD (n = 3). * p < 0.05, ** p < 0.01, *** p < 0.001: chemoresistant cells (MDR-positive) versus the corresponding chemosensitive (MDR-negative) cells.
(TIF)

S3 Fig Effects of chemotherapeutic drugs on kynurenine synthesis. A549 and A549/dx cells were incubated for 48 h in fresh medium (CTRL) or in medium containing 1 μmol/L doxorubicin (DOX), 10 μmol/L cisplatin (Pt), 100 nmol/L gemcitabine (GEM), 10 μmol/L mitoxantrone (MXR). A. Cell viability was assessed by the neutral red staining, as reported under Materials and methods. Data are presented as means ± SD (n = 4). ** p < 0.005: versus A549 CTRL cells; ° p < 0.05: A549/dx versus A549 cells. B. The kynurenine levels in the cell culture supernatants were measured spectrophotometrically. Data are presented as means ± SD (n = 4). * p < 0.01: A549/dx cells versus A549 cells.
(TIF)

S4 Fig Effects of nitric oxide on kynurenine synthesis. A549 and A549/dx cells were incubated for 24 h in the absence (CTRL) or in the presence of 100 μmol/L S-nitrosoglutathione (*GSNO*) or S-nitroso-N-acetylpenicillamine (SNAP), chosen as NO donors. **A**. The amount of nitrite in the cell culture supernatants was measured spectrophotometrically by the Griess method. Data are presented as means ± SD (n = 3). ** p < 0.005: A549- or A549/dx-treated cells versus the respective CTRL cells. B. The kynurenine levels in the cell culture supernatants were measured spectrophotometrically. Data are presented as means ± SD (n = 3). *** p < 0.001: A549/dx cells versus A549 cells.
(TIF)

S5 Fig Effects of iron on kynurenine synthesis. A549 and A549/dx cells were incubated for 24 h with fresh medium (CTRL), with the cell permeable iron-releasing compound ferric nitrilotriacetate (60 μmol/L, FeNTA) or with the iron chelator desferroxamine (100 μmol/L, DFX). **A**. The amount of intracellular iron was measured by atomic absorption spectroscopy in the cell lysate. Data are presented as means ± SD (n = 3). *** p < 0.001: A549- or A549/dx-treated cells versus the respective CTRL cells. B. The kynurenine levels in the cell culture supernatants were measured spectrophotometrically. Data are presented as means ± SD (n = 3). * p < 0.05: A549/dx cells versus A549 cells.
(TIF)

S6 Fig T-lymphocytes subclasses after co-incubation with chemosensitive and multidrug resistant cells. A549/dx cells were grown for 48 h in fresh medium (CTRL), treated with a non-targeting scrambled siRNA (scr) or with a specific siRNAs pool targeting STAT1 or STAT3, respectively (si STAT1, si STAT3). Untreated chemosensitive A549 cells were used as control. The percentage of each subclass of CD3^+^ T-lymphocytes collected from PBMC, after a 72 h co-incubation with A549 and A549/dx cells, was measured by flow cytometry. A. Percentage of T-helper (CD3^+^CD4^+^) and T-cytotoxic (CD3^+^CD8^+^) lymphocytes. Data are presented as means ± SD (n = 4). B. Percentage of Treg (CD4^+^CD25^+^CD127^low^) lymphocytes. Data are presented as means ± SD (n = 4).
(TIF)

S7 Fig Effects of IDO1 inhibition in multidrug resistant cells on the proliferation of T-lymphocytes. A. The proliferation of activated T-lymphocytes collected from PBMC after a 72 h co-incubation with A549/dx cells, grown in the absence (CTRL) or in the presence of the IDO1 inhibitor 5-Br-brassinin (100 μmol/L, BRA), was measured with the [^3^H]thymidine assay. In the presence of anti-CD3 and anti-CD28 stimulatory antibodies without tumor cells (positive control), the [^3^H]thymidine incorporation was 27,876 ± 2,349 cpm; in the presence of RPMI medium alone (negative control), the [^3^H]thymidine incorporation was 3,981 ± 705 cpm. Data are presented as means ± SD (n = 4). *** p < 0.005: BRA-treated cells versus CTRL cells. B. The percentage of CD3^+^ T-lymphocytes collected from PBMC, co-incubated with A549/dx cells as reported in A, was measured by flow cytometry. Data are presented as means ± SD (n = 4). *** p < 0.005: BRA-treated cells versus CTRL cells.
(TIF)

S1 Table PCR array of JAK/STAT-signaling genes in A549 and A549-dx cells. (DOC)

S2 Table PCR array of IL6/STAT3-signaling genes in A549 and A549-dx cells. (DOC)
