# Supplementary figures and images for: An Autocrine Cytokine/JAK/STAT-Signaling Induces Kynurenine Synthesis in Multidrug Resistant Human Cancer Cells
Source: PLoS One. 2015 May 8;10(5):e0126159. doi: 10.1371/journal.pone.0126159 (PMC4425697; doi:10.1371/journal.pone.0126159)

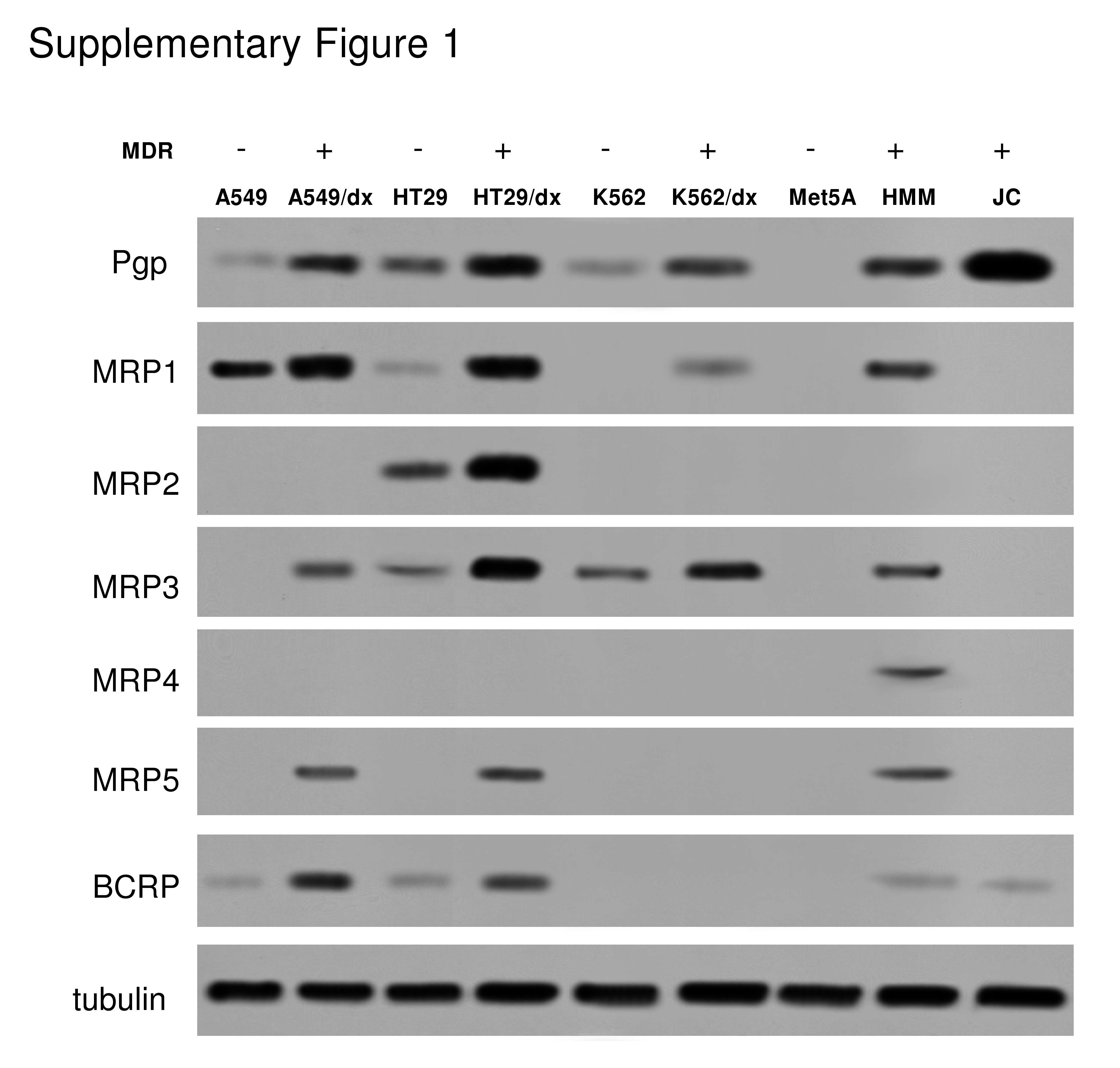

Supplement: S1 Fig — Human chemosensitive lung cancer A549 cells and chemoresistant A549/dx cells, human chemosensitive colon cancer HT29 cells and chemoresistant HT29/dx cells, human chemosensitive chronic myelogenous leukemia K562 cells and chemoresistant K562/dx cells, human chemosensitive mesothelial Met5A cells and human chemoresistant malignant mesothelioma HMM cells, murine chemoresistant mammary JC cells were lysed and subjected to the Western blot analysis for Pgp, MRP1, MRP2, MRP3, MRP4, MRP5, BCRP. The β-tubulin expression was used as control of equal protein loading. The figure is representative of 3 experiments with similar results. MDR-: chemosensitive cell lines; MDR +: chemoresistant cell lines. (TIF) [file pone.0126159.s001.tif]

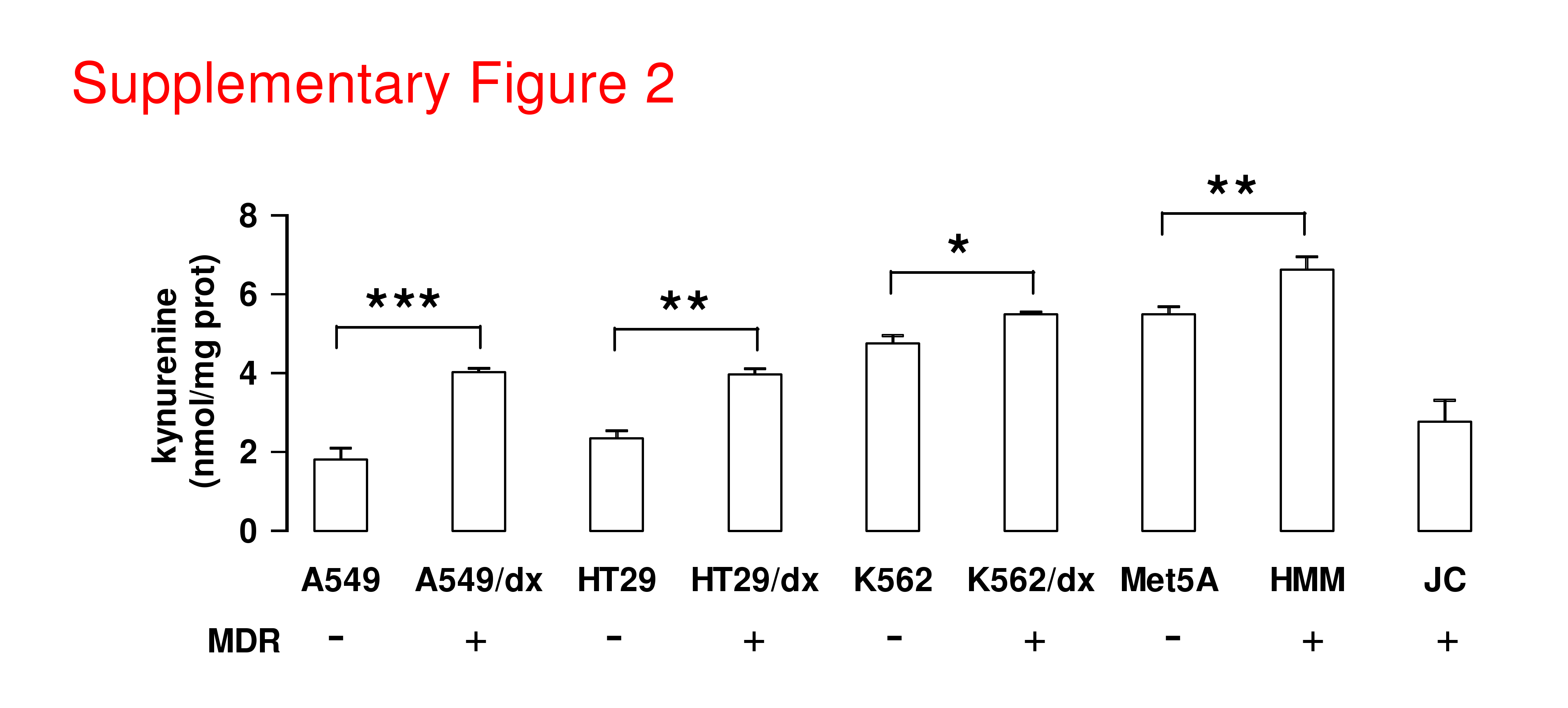

Supplement: S2 Fig — The amount of kynurenine was measured by HPLC in the culture supernatants of human chemosensitive lung cancer A549 cells and chemoresistant A549/dx cells, human chemosensitive colon cancer HT29 cells and chemoresistant HT29/dx cells, human chemosensitive chronic myelogenous leukemia K562 cells and chemoresistant K562/dx cells, human chemosensitive mesothelial Met5A cells and human chemoresistant malignant mesothelioma HMM cells, murine chemoresistant mammary JC cells. Data are presented as means ± SD (n = 3). * p < 0.05, ** p < 0.01, *** p < 0.001: chemoresistant cells (MDR-positive) versus the corresponding chemosensitive (MDR-negative) cells. (TIF) [file pone.0126159.s002.tif]

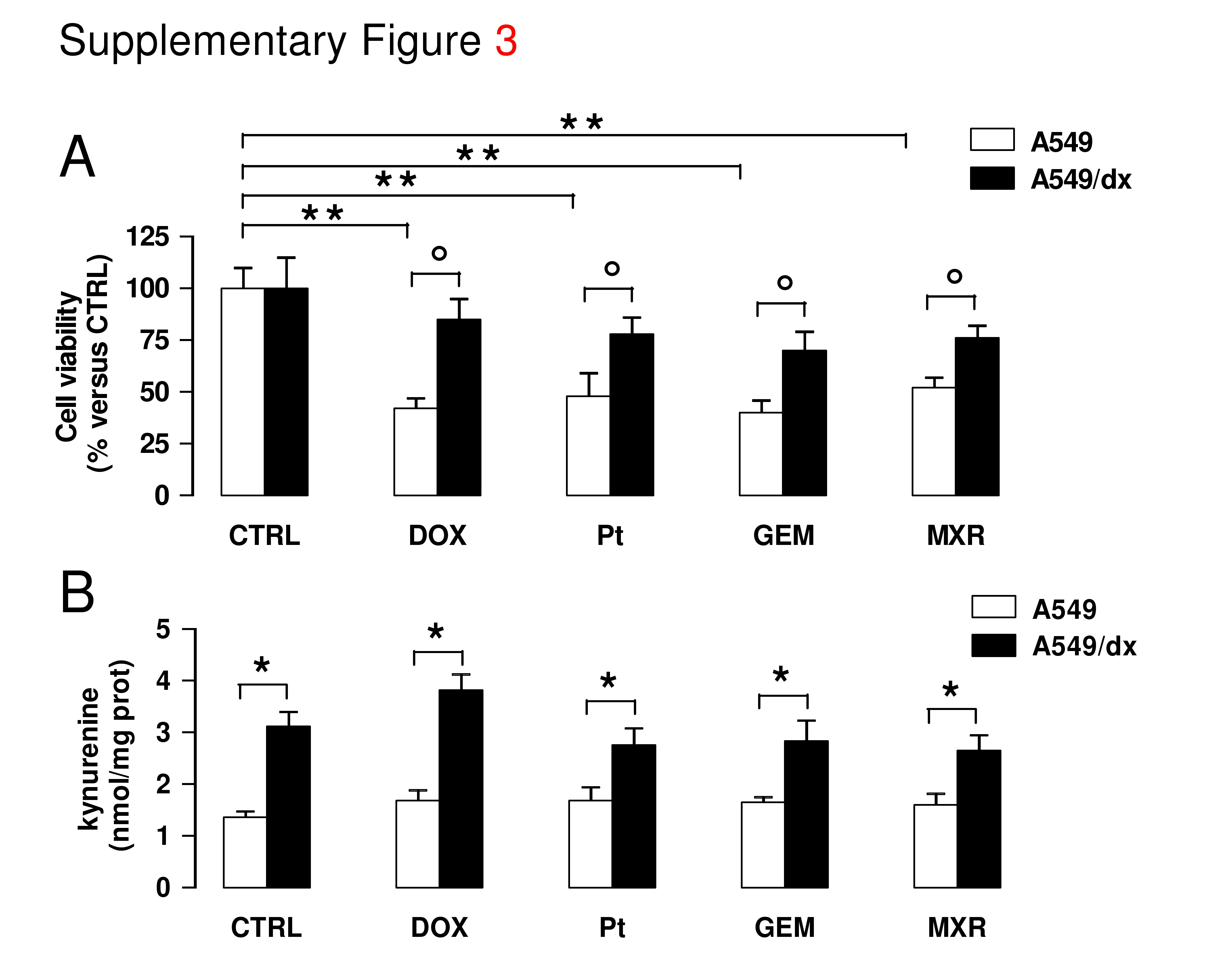

Supplement: S3 Fig — A549 and A549/dx cells were incubated for 48 h in fresh medium (CTRL) or in medium containing 1 μmol/L doxorubicin (DOX), 10 μmol/L cisplatin (Pt), 100 nmol/L gemcitabine (GEM), 10 μmol/L mitoxantrone (MXR). A. Cell viability was assessed by the neutral red staining, as reported under Materials and methods. Data are presented as means ± SD (n = 4). ** p < 0.005: versus A549 CTRL cells; ° p < 0.05: A549/dx versus A549 cells. B. The kynurenine levels in the cell culture supernatants were measured spectrophotometrically. Data are presented as means ± SD (n = 4). * p < 0.01: A549/dx cells versus A549 cells. (TIF) [file pone.0126159.s003.tif]

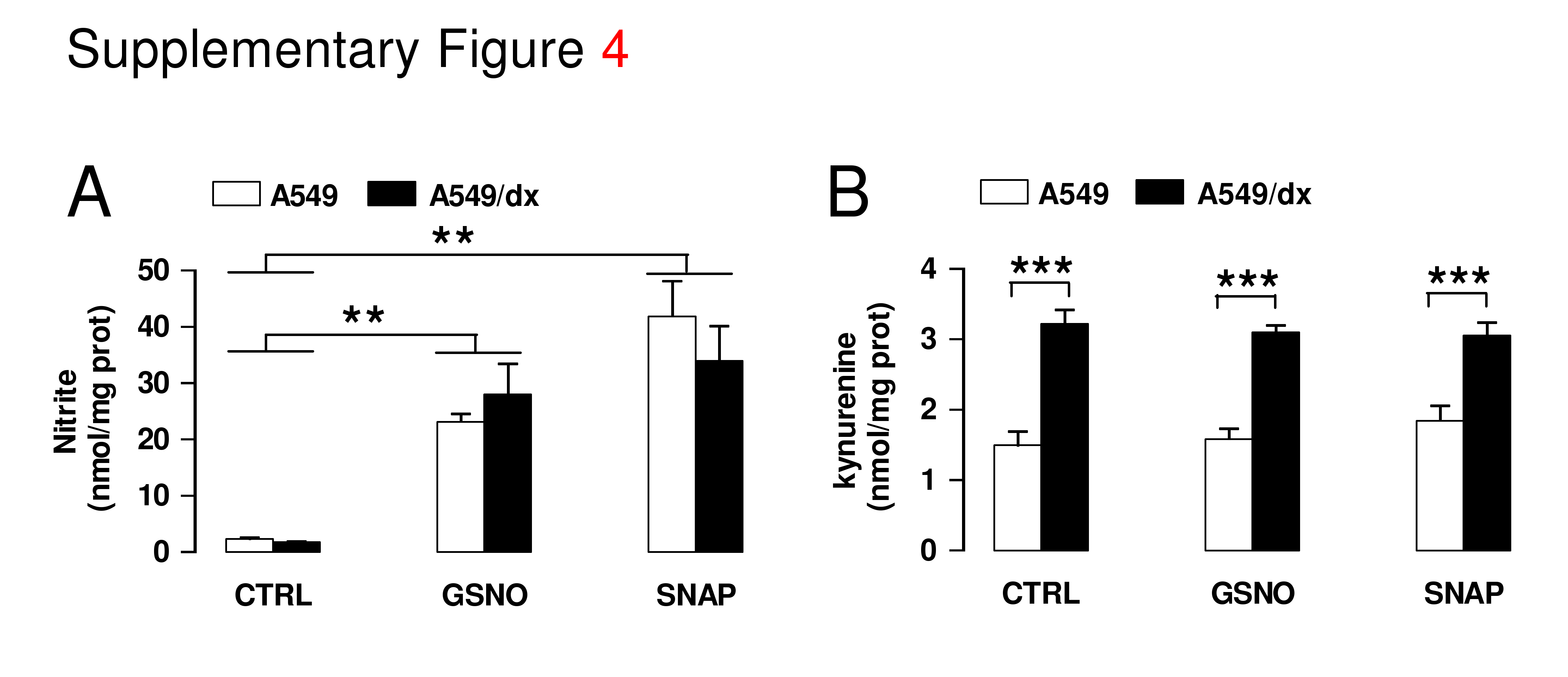

Supplement: S4 Fig — A549 and A549/dx cells were incubated for 24 h in the absence (CTRL) or in the presence of 100 μmol/L S-nitrosoglutathione (GSNO) or S-nitroso-N-acetylpenicillamine (SNAP), chosen as NO donors. A. The amount of nitrite in the cell culture supernatants was measured spectrophotometrically by the Griess method. Data are presented as means ± SD (n = 3). ** p < 0.005: A549- or A549/dx-treated cells versus the respective CTRL cells. B. The kynurenine levels in the cell culture supernatants were measured spectrophotometrically. Data are presented as means ± SD (n = 3). *** p < 0.001: A549/dx cells versus A549 cells. (TIF) [file pone.0126159.s004.tif]

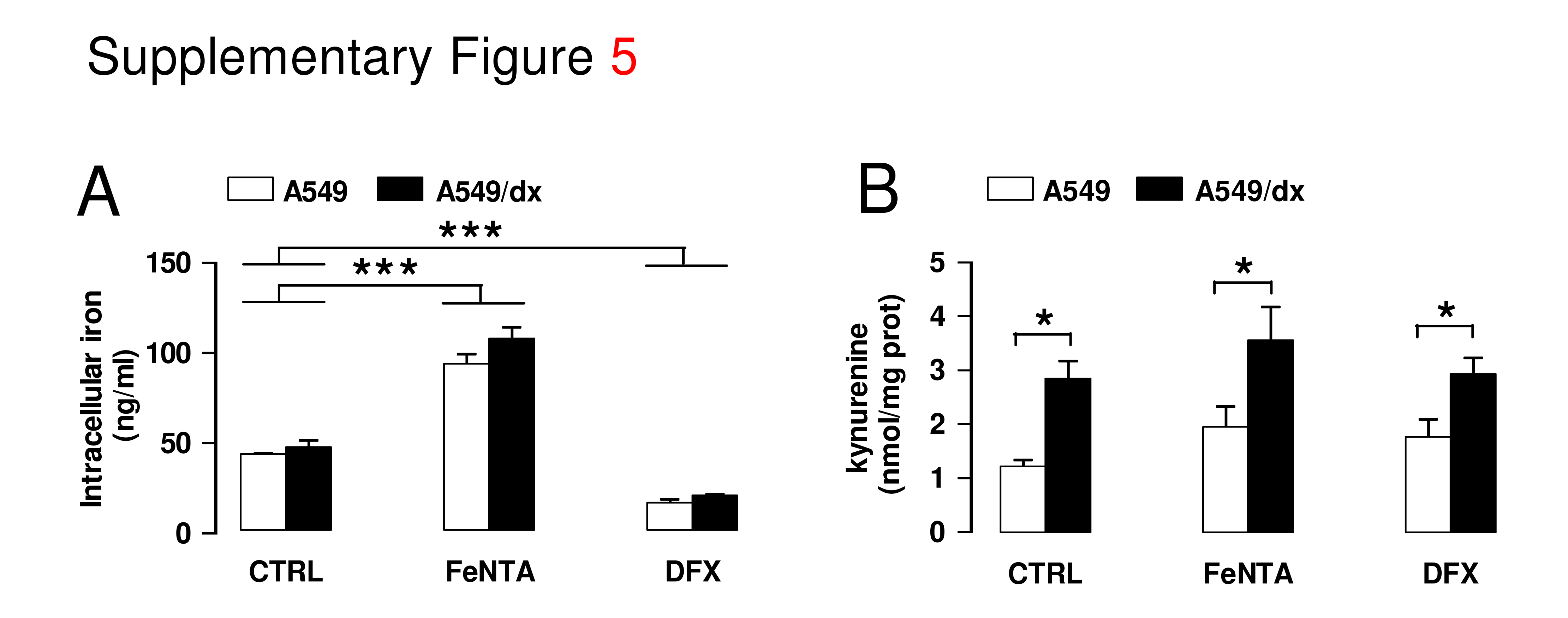

Supplement: S5 Fig — A549 and A549/dx cells were incubated for 24 h with fresh medium (CTRL), with the cell permeable iron-releasing compound ferric nitrilotriacetate (60 μmol/L, FeNTA) or with the iron chelator desferroxamine (100 μmol/L, DFX). A. The amount of intracellular iron was measured by atomic absorption spectroscopy in the cell lysate. Data are presented as means ± SD (n = 3). *** p < 0.001: A549- or A549/dx-treated cells versus the respective CTRL cells. B. The kynurenine levels in the cell culture supernatants were measured spectrophotometrically. Data are presented as means ± SD (n = 3). * p < 0.05: A549/dx cells versus A549 cells. (TIF) [file pone.0126159.s005.tif]

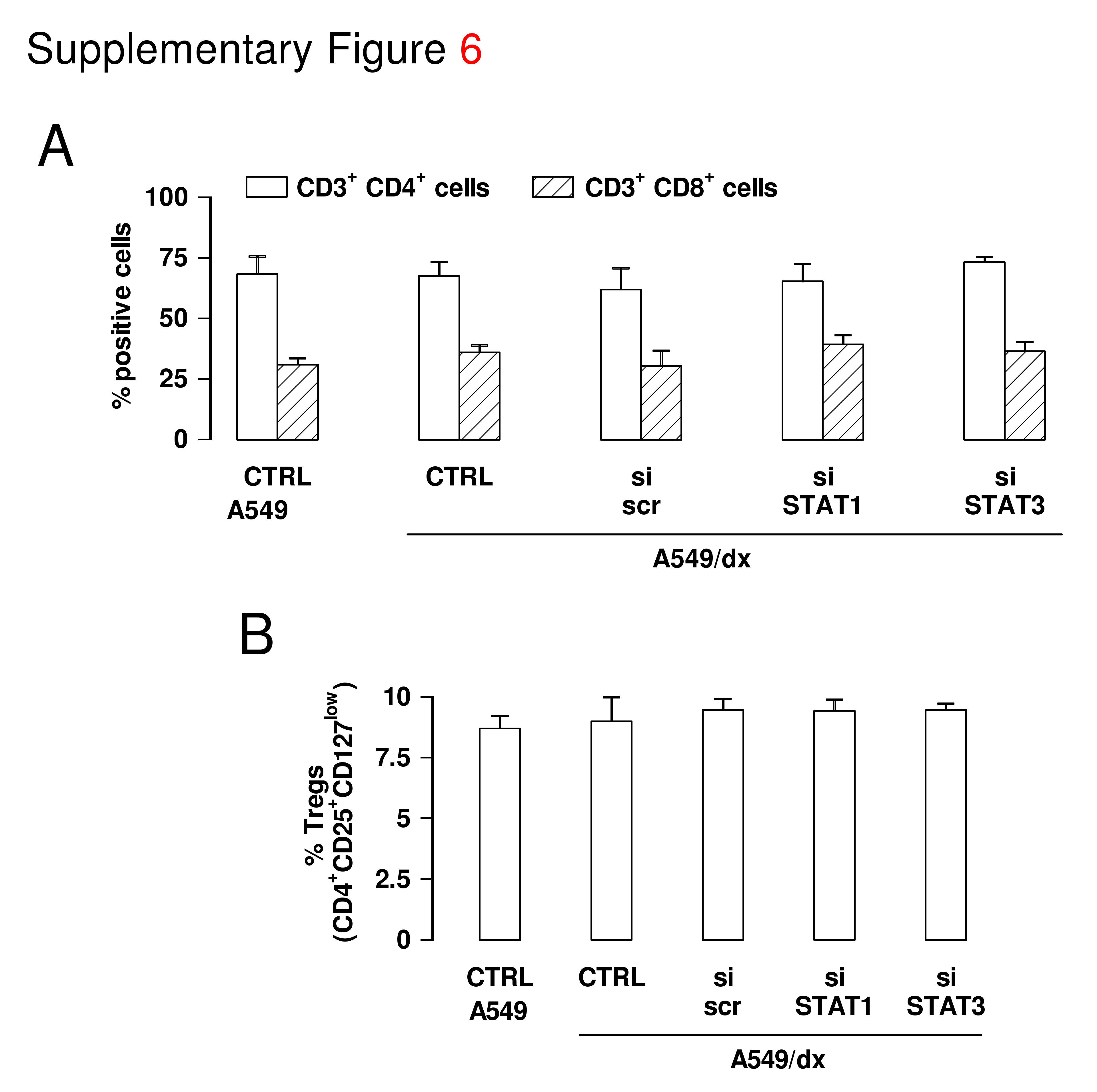

Supplement: S6 Fig — A549/dx cells were grown for 48 h in fresh medium (CTRL), treated with a non-targeting scrambled siRNA (scr) or with a specific siRNAs pool targeting STAT1 or STAT3, respectively (si STAT1, si STAT3). Untreated chemosensitive A549 cells were used as control. The percentage of each subclass of CD3+ T-lymphocytes collected from PBMC, after a 72 h co-incubation with A549 and A549/dx cells, was measured by flow cytometry. A. Percentage of T-helper (CD3+CD4+) and T-cytotoxic (CD3+CD8+) lymphocytes. Data are presented as means ± SD (n = 4). B. Percentage of Treg (CD4+CD25+CD127low) lymphocytes. Data are presented as means ± SD (n = 4). (TIF) [file pone.0126159.s006.tif]

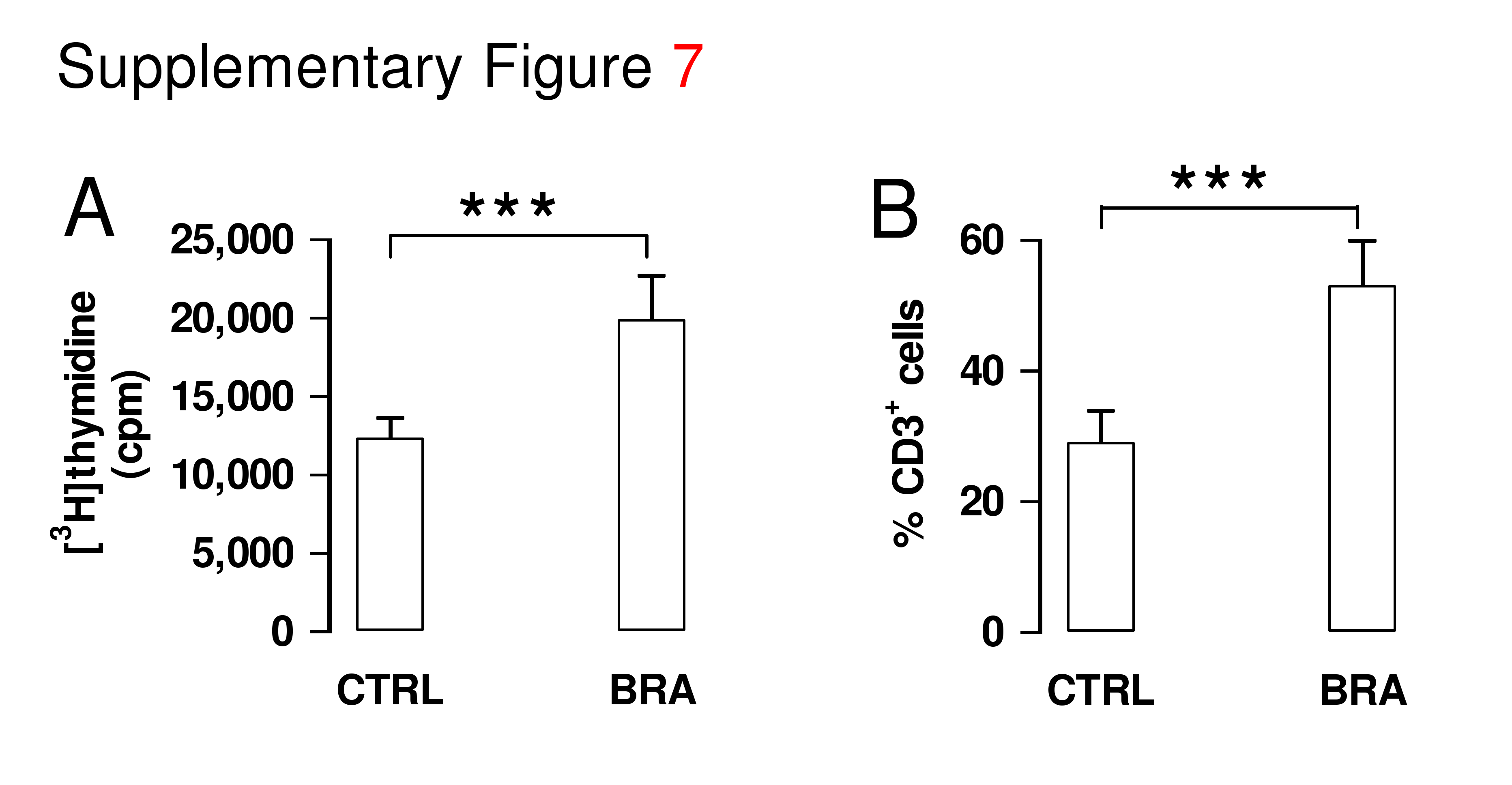

Supplement: S7 Fig — A. The proliferation of activated T-lymphocytes collected from PBMC after a 72 h co-incubation with A549/dx cells, grown in the absence (CTRL) or in the presence of the IDO1 inhibitor 5-Br-brassinin (100 μmol/L, BRA), was measured with the [3H]thymidine assay. In the presence of anti-CD3 and anti-CD28 stimulatory antibodies without tumor cells (positive control), the [3H]thymidine incorporation was 27,876 ± 2,349 cpm; in the presence of RPMI medium alone (negative control), the [3H]thymidine incorporation was 3,981 ± 705 cpm. Data are presented as means ± SD (n = 4). *** p < 0.005: BRA-treated cells versus CTRL cells. B. The percentage of CD3+ T-lymphocytes collected from PBMC, co-incubated with A549/dx cells as reported in A, was measured by flow cytometry. Data are presented as means ± SD (n = 4). *** p < 0.005: BRA-treated cells versus CTRL cells. (TIF) [file pone.0126159.s007.tif]
