# Supplementary material for: An Autocrine Cytokine/JAK/STAT-Signaling Induces Kynurenine Synthesis in Multidrug Resistant Human Cancer Cells
Source: PLoS One. 2015 May 8;10(5):e0126159. doi: 10.1371/journal.pone.0126159 (PMC4425697; doi:10.1371/journal.pone.0126159)
Supplement: S1 Table — (DOC) [file pone.0126159.s008.doc]

**Table S1**. PCR array of JAK/STAT-signaling genes in A549 and A549-dx cells

|  | **Fold change** | **Student’s t** | **Fold regulation** | **STAT1/3** |
| --- | --- | --- | --- | --- |
|  | **(A549dx vs A549)^a^** | **(p value)^b^** | **(A549dx vs A549)^c^** | **targets** |
|  | Fold change | Student’s t | Fold regulation |  |
| A2M | 2.99 | 0.002 | 2.99 | X |
| BCL2L1 | 5.03 | 0.05 | 5.03 | X |
| CCND1 | 1.00 | ns | -1.00 |  |
| CDKN1A | 3.81 | 0.01 | 3.81 | X |
| CEBPB | 1.13 | 0.0001 | 1.13 |  |
| CRK | 0.43 | 0.02 | -2.33 |  |
| CRP | 4.35 | 0.02 | 4.35 | X |
| CSF1R | 2.55 | 0.005 | 2.55 |  |
| CSF2RB | 1.13 | 0.02 | 1.13 |  |
| CXCL9 | 3.20 | 0.001 | 3.20 | X |
| EGFR | 2.83 | 0.0005 | 2.83 |  |
| EPOR | 2.11 | 0.05 | 2.11 |  |
| F2R | 1.30 | ns | 1.30 |  |
| FAS | 2.41 | ns | 2.41 | X |
| FCER1A | 0.80 | 0.0001 | -1.25 |  |
| FCGR1A | 2.11 | ns | 2.11 |  |
| GATA3 | 1.09 | ns | 1.09 |  |
| GBP1 | 2.27 | 0.050 | 2.27 |  |
| GHR | 3.68 | 0.00005 | 3.68 |  |
| HMGA1 | 0.80 | ns | -1.25 |  |
| IFNAR1 | 1.13 | ns | 1.13 |  |
| IFNG | 3.20 | 0.001 | 3.20 |  |
| IFNGR1 | 2.51 | ns | 2.51 |  |
| IL10RA | 0.35 | ns | -2.86 |  |
| IL20 | 3.20 | 0.001 | 3.20 |  |
| IL2RA | 4.23 | 0.01 | 4.23 |  |
| IL2RG | 1.60 | ns | 1.60 |  |
| IL4 | 3.20 | 0.001 | 3.20 |  |
| IL4R | 12.82 | 0.02 | 12.82 |  |
| IL6ST | 2.00 | 0.005 | 2.00 |  |
| INSR | 0.40 | ns | -2.50 |  |
| IRF1 | 3.20 | 0.05 | 3.20 | X |
| IRF9 | 6.41 | 0.0001 | 6.41 |  |
| ISG15 | 0.48 | 0.0002 | -2.08 |  |
| JAK1 | 1.13 | ns | 1.13 |  |
| JAK2 | 1.20 | ns | 1.20 |  |
| JAK3 | 2.11 | ns | 2.11 |  |
| JUN | 12.82 | 0.02 | 12.82 |  |
| JUNB | 2.73 | 0.02 | 2.73 | X |
| MMP3 | 4.53 | 0.01 | 4.53 | X |
| MPL | 0.32 | 0.002 | -3.13 |  |
| MYC | 3.12 | 0.02 | 3.12 | X |
| NFKB1 | 1.12 | ns | 1.12 |  |
| NOS2 | 2.60 | 0.02 | 2.60 | X |
| NR3C1 | 0.87 | ns | -1.15 |  |
| OAS1 | 2.60 | ns | 2.60 |  |
| OSM | 4.86 | 0.001 | 4.86 |  |
| PDGFRA | 6.63 | 0.02 | 6.63 |  |
| PIAS1 | 1.31 | ns | 1.31 |  |
| PIAS2 | 1.21 | ns | 1.21 |  |
| PPP2R1A | 1.91 | ns | 1.91 |  |
| PRLR | 2.04 | ns | 2.04 |  |
| PTPN1 | 4.53 | 0.0001 | 4.53 |  |
| PTPRC | 0.89 | ns | -1.13 |  |
| SH2B1 | 1.66 | 0.05 | 1.66 |  |
| SH2B2 | 1.02 | ns | 1.02 |  |
| SIT1 | 1.13 | ns | 1.13 |  |
| SLA2 | 0.70 | ns | -1.43 |  |
| SMAD1 | 4.53 | 0.0001 | 4.53 |  |
| SMAD2 | 0.55 | ns | -1.82 |  |
| SMAD3 | 3.68 | 0.05 | 3.68 |  |
| SMAD4 | 0.55 | ns | -1.82 |  |
| SMAD5 | 3.81 | 0.0002 | 3.81 |  |
| SOCS1 | 4.53 | 0.0001 | 4.53 | X |
| SOCS2 | 0.61 | ns | -1.64 |  |
| SOCS3 | 2.41 | 0.0001 | 2.41 |  |
| SOCS4 | 1.06 | 0.05 | 1.06 |  |
| SOCS5 | 3.20 | 0.001 | 3.20 |  |
| SP1 | 0.67 | 0.005 | -1.49 |  |
| SPI1 | 9.38 | 0.0001 | 9.38 |  |
| SRC | 4.53 | 0.05 | 4.53 |  |
| STAM | 0.46 | ns | -2.17 |  |
| STAT1 | 2.49 | 0.02 | 2.49 |  |
| STAT2 | 2.89 | 0.002 | 2.89 |  |
| STAT3 | 2.60 | 0.05 | 2.60 |  |
| STAT4 | 0.89 | 0.0001 | -1.12 |  |
| STAT5A | 0.92 | 0.05 | -1.09 |  |
| STAT5B | 1.13 | 0.01 | 1.13 |  |
| STAT6 | 0.80 | 0.05 | -1.25 |  |
| STUB1 | 12.82 | 0.05 | 12.82 |  |
| TGM2 | 2.62 | 0.01 | 2.62 |  |
| TNFRSF1A | 1.51 | 0.05 | 1.51 |  |
| TYK2 | 2.17 | ns | 2.17 |  |
| USF1 | 2.27 | 0.01 | 2.27 |  |
| YY1 | 0.15 | 0.001 | -6.67 |  |
| TUBB2A | 1.17 | ns | 1.17 |  |
| ACTB | 1.03 | ns | 1.03 |  |
| B2M | 1.05 | ns | 1.05 |  |
| GAPDH | 1.03 | ns | 1.03 |  |
| HPRT1 | 1.21 | ns | 1.21 |  |

^a^ Fold-Change (2^(- Delta Delta Ct)) is the normalized gene expression (2^(- Delta Ct)) in A549/dx cells divided by the normalized gene expression (2^(- Delta Ct)) in A549 cells (n= 4) where Ct is the threshold cycle in qRT-PCR; fold-change values greater than 1 indicate an up-regulation, fold-change values less than 1 indicate a down-regulation.

^b^ The p values are calculated based on a Student’s t-test of the replicate 2^(- Delta Ct) values for each gene in A549 cells and A549/dx cells; p < 0.05 was considered significant. ns: not significant.

^c^ Fold-Regulation represents fold-change results in a biologically meaningful way: when fold-change values are greater than 1 the fold-regulation is equal to the fold-change, when fold-change values are less than 1 the fold regulation is the negative inverse of the fold-change.
