# Supplementary material for: An Autocrine Cytokine/JAK/STAT-Signaling Induces Kynurenine Synthesis in Multidrug Resistant Human Cancer Cells
Source: PLoS One. 2015 May 8;10(5):e0126159. doi: 10.1371/journal.pone.0126159 (PMC4425697; doi:10.1371/journal.pone.0126159)
Supplement: S2 Table — (DOC) [file pone.0126159.s009.doc]

**Table S2**. PCR array of IL6/STAT3-signaling genes in A549 and A549-dx cells

|  | **Fold change** | **Student’s t** | **Fold regulation** | **IDO** |
| --- | --- | --- | --- | --- |
|  | **(A549dx vs A549)^a^** | **(p value)^b^** | **(A549dx vs A549)^c^** | **inducers** |
|  | Fold change | Student’s t | Fold regulation |  |
| AKT1 | 3.12 | 0.05 | 3.12 |  |
| BAX | 1.85 | Ns | 1.85 |  |
| BCL2 | 2.81 | 0.05 | 2.81 |  |
| BCL3 | 1.56 | ns | 1.56 |  |
| CASP4 | 2.62 | 0.01 | 2.62 |  |
| CCL2 | 0.42 | 0.05 | -2.38 |  |
| CCL3 | 1.56 | ns | 1.56 |  |
| CCL4 | 2.62 | 0.01 | 2.62 |  |
| CCL5 | 2.62 | 0.01 | 2.62 |  |
| CD4 | 2.62 | 0.01 | 2.62 |  |
| CD40 | 1.99 | 0.000005 | 1.99 |  |
| CD40LG | 2.28 | 0.02 | 2.28 | X |
| CDKN1A | 3.36 | 0.02 | 3.36 |  |
| CEBPD | 2.91 | ns | 2.91 |  |
| CSF1 | 2.62 | 0.01 | 2.62 |  |
| CSF2 | 2.13 | 0.02 | 2.13 |  |
| CSF3 | 2.71 | 0.01 | 2.71 |  |
| CSF3R | 2.62 | 0.01 | 2.62 |  |
| CXCL10 | 2.13 | 0.02 | 2.13 |  |
| EGFR | 2.62 | 0.01 | 2.62 |  |
| FAS | 2.62 | 0.01 | 2.62 |  |
| FASLG | 2.13 | 0.01 | 2.13 |  |
| GLRX | 2.28 | 0.008859 | 2.28 |  |
| HGF | 2.62 | 0.01 | 2.62 |  |
| IFITM2 | 2.62 | 0.009028 | 2.62 |  |
| IL10 | 2.62 | 0.01 | 2.62 |  |
| IL11 | 2.62 | 0.01 | 2.62 |  |
| IL13 | 2.28 | 0.01 | 2.28 | X |
| IL15 | 1.40 | Ns | 1.40 |  |
| IL17A | 2.62 | 0.01 | 2.62 |  |
| IL18 | 1.67 | 0.01 | 1.67 |  |
| IL18R1 | 2.62 | 0.01 | 2.62 |  |
| IL1A | 2.62 | 0.009028 | 2.62 |  |
| IL1B | 2.62 | 0.009028 | 2.62 | X |
| IL1R1 | 2.13 | 0.02 | 2.13 |  |
| IL2 | 2.62 | 0.01 | 2.62 |  |
| IL21 | 2.62 | 0.01 | 2.62 |  |
| IL22 | 1.99 | 0.0001 | 1.99 |  |
| IL23A | 2.62 | 0.01 | 2.62 |  |
| IL2RA | 4.11 | 0.0001 | 4.11 |  |
| IL3 | 2.62 | 0.01 | 2.62 |  |
| IL4 | 3.47 | 0.01 | 3.47 | X |
| IL5 | 2.62 | 0.01 | 2.62 |  |
| IL6 | 6.92 | 0.00002 | 6.92 | X |
| IL6R | 2.62 | 0.01 | 2.62 |  |
| IL6ST | 1.85 | 0.005 | 1.85 |  |
| IL8 | 3.71 | 0.039155 | 3.71 |  |
| JAK2 | 1.56 | ns | 1.56 |  |
| JAK3 | 2.51 | ns | 2.51 |  |
| JUNB | 2.62 | 0.01 | 2.62 |  |
| LIF | 2.91 | 0.0001 | 2.91 |  |
| LIFR | 2.62 | 0.01 | 2.62 |  |
| LTA | 2.62 | 0.01 | 2.62 |  |
| MAP2K1 | 1.85 | 0.0001 | 1.85 |  |
| MAPK1 | 3.71 | 0.05 | 3.71 |  |
| MAPK14 | 1.56 | ns | 1.56 |  |
| MAPK3 | 1.51 | ns | 1.51 |  |
| MAPK8 | 1.56 | ns | 1.56 |  |
| MET | 1.85 | ns | 1.85 |  |
| MTOR | 2.62 | 0.01 | 2.62 |  |
| MYC | 3.53 | 0.002 | 3.53 |  |
| NFKB1 | 1.56 | ns | 1.56 |  |
| NFKBIA | 1.99 | ns | 1.99 |  |
| NRP1 | 3.97 | 0.001 | 3.97 |  |
| OSM | 3.62 | 0.002 | 3.86 |  |
| PHF21A | 1.22 | ns | 1.22 |  |
| PIAS3 | 0.66 | 0.0001 | - 1.51 |  |
| PIM1 | 2.62 | 0.01 | 2.62 |  |
| PROS1 | 2.62 | 0.01 | 2.62 |  |
| PVRL2 | 2.20 | 0.02 | 2.20 |  |
| RELA | 1.56 | ns | 1.56 |  |
| SOCS1 | 4.12 | ns | 4.12 |  |
| SOCS3 | 2.12 | 0.05 | 2.12 |  |
| SRC | 4.73 | 0.05 | 4.73 |  |
| STAT3 | 2.62 | 0.01 | 2.62 |  |
| TGM2 | 2.62 | 0.01 | 2.62 |  |
| TLR4 | 3.34 | 0.01 | 3.34 |  |
| TNF | 2.62 | 0.01 | 2.62 | X |
| TNFRSF10B | 1.73 | 0.001 | 1.73 |  |
| TNFRSF1A | 1.61 | 0.05 | 1.61 |  |
| TNFRSF1B | 2.62 | 0.01 | 2.62 |  |
| TNFSF10 | 2.62 | 0.01 | 2.62 |  |
| TYK2 | 2.62 | 0.01 | 2.62 |  |
| TUBB2A | 1.02 | ns | 1.02 |  |
| ACTB | 1.15 | ns | 1.15 |  |
| B2M | 1.13 | ns | 1.13 |  |
| GAPDH | 1.27 | ns | 1.27 |  |
| HPRT1 | 1.08 | ns | 1.08 |  |

^a^ Fold-Change (2^(- Delta Delta Ct)) is the normalized gene expression (2^(- Delta Ct)) in A549/dx cells divided by the normalized gene expression (2^(- Delta Ct)) in A549 cells (n= 4) where Ct is the threshold cycle in qRT-PCR; fold-change values greater than 1 indicate an up-regulation, fold-change values less than 1 indicate a down-regulation.

^b^ The p values are calculated based on a Student’s t-test of the replicate 2^(- Delta Ct) values for each gene in A549 cells and A549/dx cells; p < 0.05 was considered significant. ns: not significant.

^c^ Fold-Regulation represents fold-change results in a biologically meaningful way: when fold-change values are greater than1 the fold-regulation is equal to the fold-change, when fold-change values are less than 1 the fold regulation is the negative inverse of the fold-change.
